# Synergistic Single-Atom and Clustered Cobalt Sites on N/S Co-Doped Defect Nano-Carbon for Efficient H_2_O_2_ Electrosynthesis

**DOI:** 10.1007/s40820-025-01657-9

**Published:** 2025-02-12

**Authors:** Yuzhong Huang, Chang Zhang, Xingyu Wang, Yuji Wu, Jun Lv, Jian Zhang, Wangqiang Shen, Xing Lu

**Affiliations:** 1 School of Materials Science and Engineering, Hefei University of Technology, Hefei, 230009 People’s Republic of China; 2 School of Materials Science and Engineering, Huazhong University of Science and Technology, Wuhan, 430074 People’s Republic of China; 3 School of Nuclear Engineering, Rocket Force University of Engineering, Xi’an, 710025 People’s Republic of China; 4 Performance Copper Alloy Materials and Processing, Ministry of Education, Engineering Research Center of High, Hefei University of Technology, Hefei, 230009 People’s Republic of China

**Keywords:** Non-noble metal-based materials, Fullerene, Single atomic catalysts, Oxygen reduction reaction, Hydrogen peroxide electroproduction

## Abstract

Non-noble-based single atomic catalysts have exhibited significant potential in electrochemical production of H_2_O_2_ via two-electron oxygen reduction reactions (2e^−^ ORR). However, constructing highly efficient and acid-resistant catalysts remains a challenge but significant. In this work, fullerene (C_60_) with abundant pentagonal inherent defects was employed as a carbon substrate to synthesize defect-rich nanocarbon electrocatalysts doped with NSCo single atoms and accompanied by metallic Co nanoparticles (CoSA/CoNP-NSDNC) for the first time. The electrochemical experiments demonstrate that the active sites of CoSA/CoNP-NSDNC are formed through the synergistic interaction between NSCo single atoms and Co nanoparticle clusters embedded within the carbon framework. The obtained CoSA/CoNP-NSDNC catalyst exhibits an onset potential as 0.72 V versus RHE and achieves up to 90% H_2_O_2_ selectivity over a wide potential range of 500 mV. Moreover, the as-obtained CoSA/CoNP-NSDNC configured as the cathode in a self-assembled flow cell under acidic conditions achieves a high H_2_O_2_ production rate of 4206.96 mmol g_cat_⁻^1^ h⁻^1^ with a Faraday efficiency of ∼ 95% and exhibit ultra fast degradation of organic pollutants. This work focuses on the synergistic effect of non-noble metal nanoparticles, metal single-atom sites, and topological defects on the 2e^−^ ORR process, which provides a new direction for designing carbon-based catalysts for efficient H_2_O_2_ electrosynthesis.

**Supplementary Information:**

The online version contains supplementary material available at 10.1007/s40820-025-01657-9.

## Highlights

- Defect-rich nanocarbon catalyst (CoSA/CoNP-NSDNC) synthesized using NSCo single atoms and Co nanoparticle clusters on fullerene-derived carbon framework, enabling efficient H_2_O_2_ electrosynthesis.
- The CoSA/CoNP-NSDNC catalyst exhibits high H_2_O_2_ selectivity (~ 90%) over a wide potential range with an onset potential of 0.72 V versus RHE, achieving Faraday efficiency close to 95% in acidic conditions.
- Demonstrates potential for environmental applications, achieving high H_2_O_2_ production (4206.96 mmol g^−1^ h^−1^) in a flow cell setup, along with efficient degradation of organic pollutants in Fenton-like reactions.

**Supplementary Information:**

The online version contains supplementary material available at 10.1007/s40820-025-01657-9.

## Graphical abstract

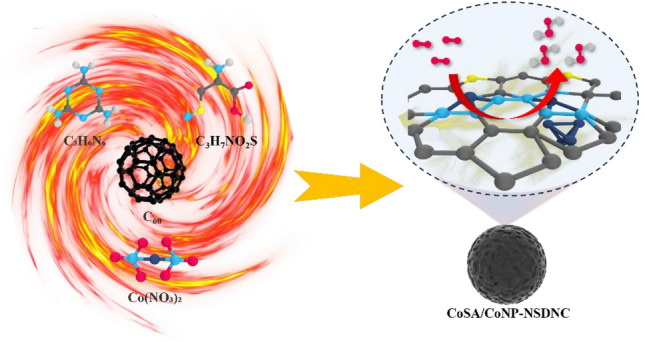

## Introduction

Hydrogen peroxide (H_2_O_2_) plays a vital role in various industries, particularly in environmental protection and sustainable development. Currently, over 98% of H_2_O_2_ is synthesized through the anthraquinone process, which is energy-intensive and generates significant organic waste [1, 2]. Additionally, the concentration of industrially produced H_2_O_2_ must be increased to 70 wt% to reduce storage and transportation costs. However, H_2_O_2_’s propensity to decompose easily introduces potential safety risks during these processes [3–6]. Therefore, the advancement of energy-saving, green, safe, and efficient H_2_O_2_ synthesis methods is of importance. Electrochemical oxygen reduction offers low energy consumption and produces clean, pollution-free products, making it a highly promising method for H_2_O_2_ production, which has attracted significant research attention [7–10]. The development of efficient two-electron (2e^−^) electrocatalysts is essential for advancing this technology. However, despite considerable progress in alkaline conditions, many electrocatalysts still exhibit limited selectivity and H_2_O_2_ yield under acidic conditions.

Noble metal catalysts (e.g., Pt, Au) have shown exceptional performance in H_2_O_2_ production [10–12]. However, these noble metals are costly and gradually lose activity due to continuous corrosion in acidic electrolytes, presenting a significant challenge for their sustainable use in catalytic applications [13, 14]. Fullerene (C_60_) is a novel carbon material with inherent pentagonal topological defects, which endows it with high electron affinity and excellent electron transfer capability. Mu et al*.* demonstrated that alkali etching of C_60_ can produce pentagon-rich carbon materials, which exhibit excellent electrochemical properties, highlighting the advantages of fullerenes in the oxygen reduction reactions (ORR) field [15]. Therefore, fullerene-based catalysts obtained by pyrolysis and derivatives are one of the most promising carbon-based materials for ORR electrocatalysts due to their high defect density and heteroatom doping potential [16]. Despite these benefits, unmodified carbon materials lacking surface functionalization generally display low activity toward 2e^− ^ORR, making it important to improve their performance through doping and the incorporation of defects [17, 18]. For example, studies have revealed that the performance of carbon-based electrocatalysts is influenced by their electronic properties, which can be modulated through heteroatom doping (e.g., N, S, and B) and the introduction of defects (e.g., edge or topological defects) [16, 19–22]. At present, non-noble metal-doped carbon electrocatalysts have been widely explored in the field of 4e^−^ ORR, with performance that even surpasses some noble metal catalysts [23, 24]. Unfortunately, for the 2e^−^ ORR process, their activity and selectivity are still unsatisfactory. On the one hand, the reaction kinetics of this process is slower than that of the 4e^−^ ORR pathway [1, 3, 5, 6, 25]. On the other hand, traditional pyrolytic synthesis methods inevitably introduce metal nanoparticles, the presence of which is often considered useless or even harmful to the 2e^−^ ORR process [26, 27]. Recently, it has been confirmed that metal atoms and metal nanoparticles introduced through pyrolysis can exhibit a synergistic catalytic effect, even exceeding the performance of carbon catalysts only doped with single atoms or metal nanoparticles [28]. Consequently, it is expected that simultaneously loading metal single atoms and metal nanoparticles onto an appropriate carbon matrix could enhance the activity and selectivity, thereby achieving higher H_2_O_2_ yields.

Herein, in this work, we visualize the synergistic effect by construct a defect-rich nanocarbon electrocatalysts with NSCo single atoms and metal nanoparticle clusters (CoSA/CoNP-NSDNC). The CoSA/CoNP-NSDNC catalyst, synthesized through a one-step pyrolysis approach, contains abundant topological defects and demonstrates remarkable ORR activity and high selectivity for H_2_O_2_. This performance is enhanced by its multiporous architecture, extensive surface area, significant topological defects, and consistent NSCo single-atom doping combined with metal nanoparticles. When CoSA/CoNP-NSDNC was used as the cathode in a flow cell electrolyzer, a remarkable H_2_O_2_ electrosynthesis capability (4206.96 mmol g_cat_⁻^1^ h⁻^1^) was observed, along with a high Faradaic efficiency (~ 95%). Such excellent 2e^−^ ORR performance under acidic conditions is comparable to that of platinum and other noble metal-based catalysts. Additionally, it demonstrates excellent degradation ability toward organic dyes in a Fenton-like system. This study aids in exploring the synergistic effect between topological defects of carbon materials and non-noble metals, which lays a foundation for designing novel carbon-based electrocatalysts to promote acidic H_2_O_2_ electrosynthesis.

## Experimental Section

### Materials

Fullerene (99.9%), melamine, L-cysteine, cobalt nitrate hexahydrate, perchloric acid, ethanol, isopropanol, sulfuric acid, ferric sulfate heptahydrate, cerium sulfate, NaSCN, malachite green, methyl blue, and Nafion (5 wt%) are all from Sigma Aldrich Reagent Co., Ltd. Carbon fiber paper (CFP) was purchased from Toray Plastic Precision Co., Ltd. Commercial titanium-based IrO_2_-coated electrode was purchased from Siotech Industrial Technology Co., Ltd. All chemical reagents are directly used in the experiment without further purification.

### Preparation of CoSA/CoNP-NSDNC and Co-NSC

#### Preparations of the CoSA/CoNP-NSDNC

0.1 g of cobalt nitrate hexahydrate was dissolved in pure water to obtain a cobalt nitrate hexahydrate solution. 0.2 g of fullerene was dissolved CS_2_, and then 4 g of melamine and 0.4 g of L-cysteine were mixed, and poured into CS_2_ containing fullerene. The mixture was heated in a water bath at 60 °C until CS_2_ was completely volatilized, and then cobalt nitrate hexahydrate solution was added. After ultrasonication for 20 min, the mixture was dried to obtain a mixture precursor. The mixture precursor obtained was heated in an argon atmosphere. In the first stage, heat it at a rate of 10 °C per minute until the temperature reaches 600 °C and then keep it at this temperature for 1 h. Subsequently, continue to heat it at a rate of 10 °C per minute until the temperature reaches 800 ~ 1100 °C and maintain this temperature for 2 h. After that, allow it to cool down to room temperature along with the furnace and then take it out. Subsequently, the acid leaching is done by heating 0.5 M H_2_SO_4_ solution at 60 °C for 8 h. The samples were washed to pH = 7, and dried under vacuum conditions to obtain a black powder, defined as CoSA/CoNP-NSDNC. For comparison, the mixture precursor was annealed at 800–1100 °C in an argon atmosphere, and the prepared product was defined as CoSA/CoNP-NSDNC-X (X = 800–1100 °C).

#### Preparations of the Co-NSC

Similarly, except for the removal of fullerene in Sect. 2.2.1 and the second stage at 1000 °C for 2 h, the other conditions remain unchanged, and the product is defined as Co-NSC.

## Results and Discussion

### Design Principle and Structural Characterizations

**Fig. 1:**
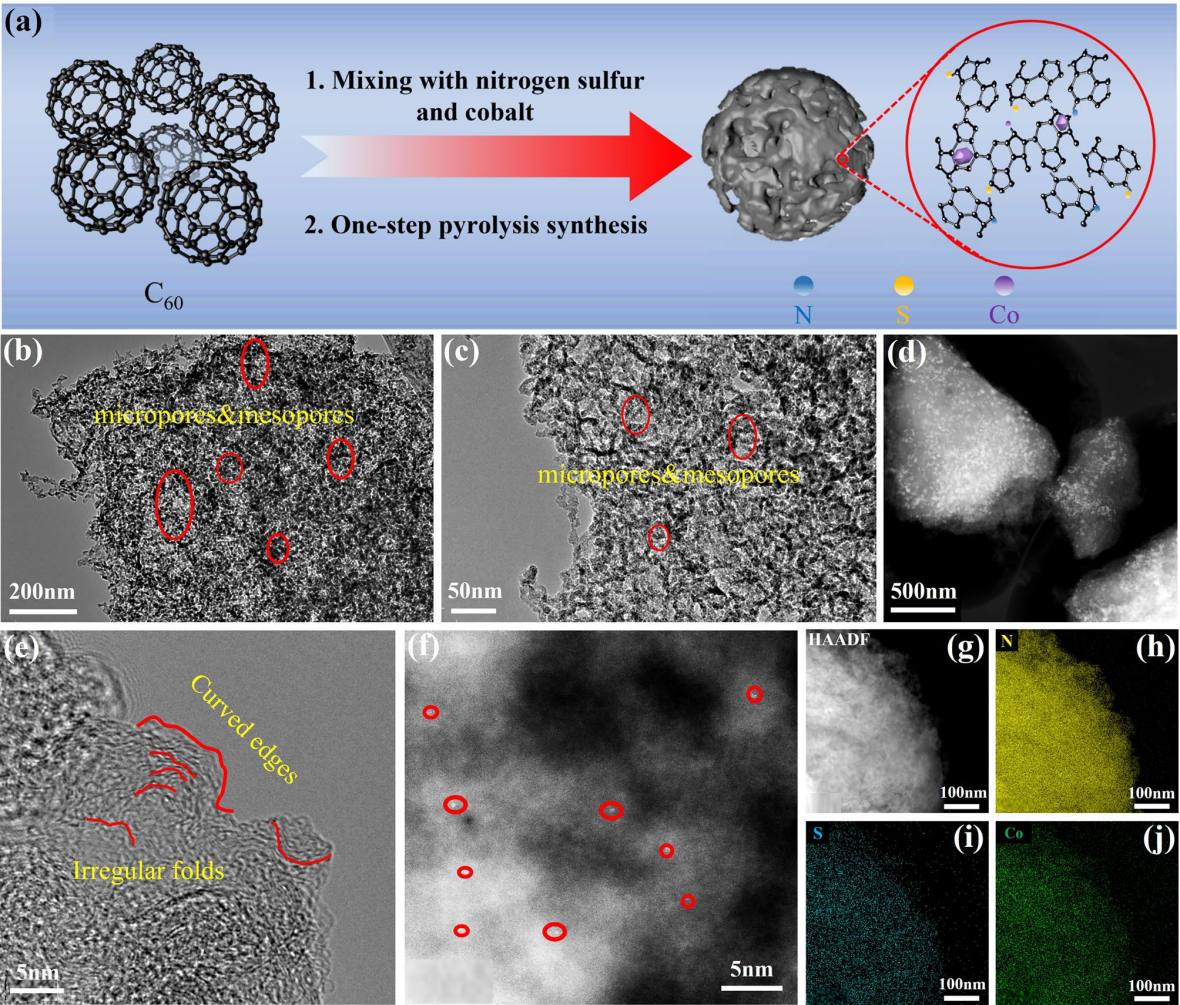
**Morphological characterization of CoSA/CoNP-NSDNC**. **a** Schematic illustration of the CoSA/CoNP-NSDNC synthesis; **b, c** TEM images of the CoSA/CoNP-NSDNC; **d-f** HR-TEM images of the CoSA/CoNP-NSDNC, with the circle in red for Co single atom; **g-j** HAADF-STEM and EDS elemental mapping for N, S, and Co of the as-obtained CoSA/CoNP-NSDNC. (Color figure online)

Fullerene is chosen as the precursor owing to its unique molecular structure with pentagonal topological defects, which provides high electron affinity and excellent electron transport properties. Additionally, the modifiable carbon cage structure of C_60_ allows for the doping of heteroatoms and the introduction of metal single atoms or clusters, effectively creating active sites with tunable electronic properties. Furthermore, compared to noble metal catalysts, C_60_-based electrocatalysts exhibit superior durability and corrosion resistance, particularly under acidic conditions. Figure S1 shows the selected area electron diffraction (SAED) and X-ray diffraction (XRD) of the original fullerene (C_60_) framework and structure. The electron diffraction pattern in Fig. S1a and the XRD pattern in Fig. S1b reveal the typical face-centered cubic (FCC) structure of fullerene. As shown in Fig. 1a, the synthesis route of the NSCo single-atom sites and adjacent cluster metal nanoparticles fullerene-based catalyst involves a two-step heating process, resulting in the one-step pyrolysis synthesis of NSCo single-atom sites and adjacent cluster metal nanoparticles defect carbon nanomaterials (CoSA/CoNP-NSDNC). The detailed synthesis methods can be found in the Supporting Information. After high-temperature pyrolysis and doping with nitrogen, sulfur, and cobalt heteroatoms, CoSA/CoNP-NSDNC exhibits an amorphous carbon structure and a loose porous morphology (Fig. S2a).

To further observe the micromorphology of CoSA/CoNP-NSDNC, transmission electron microscopy (TEM) was conducted. Specifically, the TEM images in Fig. 1b, c show that the CoSA/CoNP-NSDNC catalyst possesses a hierarchical porous structure with micropores, mesopores, and macropores. Hierarchical porous structures (including micropores, mesopores, and macropores) significantly enhance the mass transfer efficiency of reactants (e.g., O_2_ molecules) on the catalyst surface. Macropores act as “transport channels,” enabling the rapid delivery of reactants to the catalytic active sites. Mesopores serve as “dispersion zones,” mitigating local concentration polarization. Meanwhile, micropores increase the residence time of reactants near active sites, further improving catalytic efficiency [29]. Additionally, the catalyst has porous structure, which means more active sites can be exposed, thereby enhancing the electrocatalytic performance. Furthermore, the high-resolution transmission electron microscope (HR-TEM) in Fig. 1d shows the carbon skeleton contains a large number of Co particles. The irregular folds and curved edges (Fig. 1e) of CoSA/CoNP-NSDNC confirm the amorphous structure of the carbon substrate and indicate the presence of numerous topological defects due to the reconstruction of the distorted lattice during high-temperature pyrolysis [21]. The high-magnification dark-field image of the HR-TEM in Fig. 1f shows numerous distinct bright white spots embedded within the carbon matrix, suggesting that Co atoms are distributed throughout the carbon framework. The EDS images in Fig. 1g-j exhibit the uniform distribution of N, S, and Co in CoSA/CoNP-NSDNC, indicating that the heteroatoms have been successfully incorporated into the carbon substrate. These results show that the CoSA/CoNP-NSDNC possesses an amorphous structure, hierarchical porosity, and uniform heteroatom distribution. For comparison, we synthesized Co-NSC carbon materials using melamine, L-cysteine, and cobalt nitrate hexahydrate without fullerene. Contrastingly, Co-NSC exhibits fewer porous structures.

### Interaction Characterization and Mechanical Properties

**Fig. 2:**
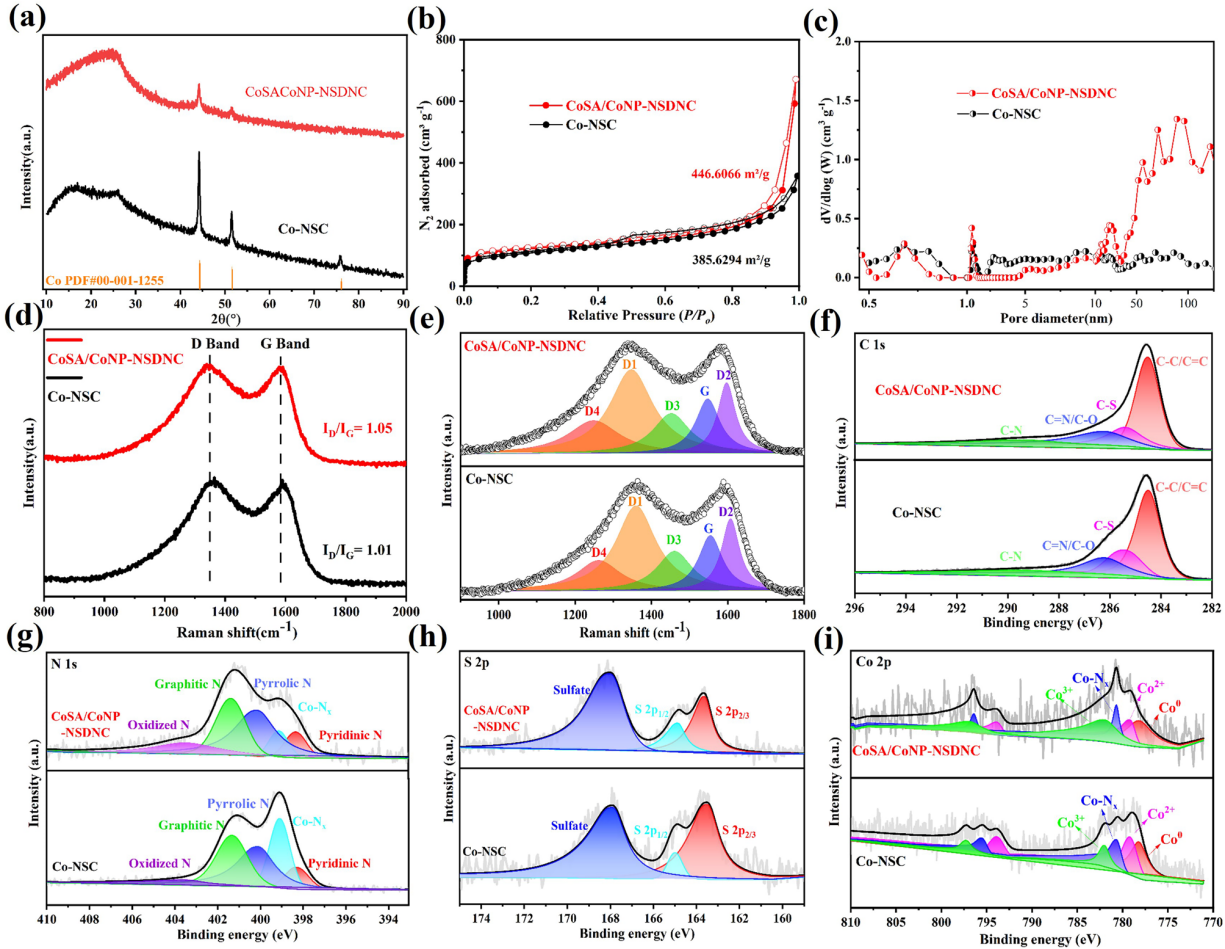
**Structural investigation of CoSA/CoNP-NSDNC and Co-NSC**. **a** XRD of the CoSA/CoNP-NSDNC and Co-NSC; **b** N_2_ adsorption and desorption isotherms of the CoSA/CoNP-NSDNC and Co-NSC; **c** pore size distribution diagram of CoSA/CoNP-NSDNC and Co-NSC; **d, e** Raman spectra of CoSA/CoNP-NSDNC and Co-NSC; XPS profile of CoSA/CoNP-NSDNC and Co-NSC and their corresponding deconvoluted XPS for **f** C 1*s*, **g** N 1*s*, **h** S 2*p*, and **i** Co 2*p* spectra

To better highlight the structural differences between CoSA/CoNP-NSDNC and Co-NSC, XRD, N_2_ adsorption–desorption isotherms, Raman spectroscopy, and X-ray photoelectron spectroscopy (XPS) were performed. These analyses revealed the samples’ porosity characteristics, defect types, and surface chemical states. The XRD patterns (Figs. 2a and S2b) reveal diffraction peaks around 26° for CoSA/CoNP-NSDNC and Co-NSC, corresponding to the (002) plane of carbon, indicating the presence of graphitic structures in the materials [30]. The (002) diffraction peak of CoSA/CoNP-NSDNC exhibits a wider half-width, suggesting a larger interlayer spacing and a higher level of amorphousness relative to Co-NSC [31]. Additionally, CoSA/CoNP-NSDNC samples exhibit two metal Co diffraction peaks at 44.2° and 51.5°, attributed to the formation of Co particles due to the strong reducing Ar plasma formed at high temperatures. Subsequently, acid washing was performed on CoSA/CoNP-NSDNC to remove unstable Co particles. The XRD pattern (Fig. S3a) still shows characteristic peaks of Co particles, and the electrochemical performance (Fig. S3b) post-washing showed no change in current. These results reveal that Co particles prepared by pyrolysis method can stably exist within the carbon framework, which may work together with Co single atoms to further enhance the corresponding catalytic performance. Figure 2b exhibits the nitrogen adsorption–desorption isotherms of CoSA/CoNP-NSDNC and Co-NSC. The isotherm of CoSA/CoNP-NSDNC shows a combination of type I and IV with an obvious hysteresis loop, indicating a hierarchical multiporous structure with a Brunauer–Emmett–Teller (BET) surface area of 466.61 m^2^g⁻^1^, higher than that of Co-NSC. The pore size distribution curves in Fig. 2c reveal that CoSA/CoNP-NSDNC possesses a hierarchical porous structure comprising micropores, mesopores, and macropores, whereas Co-NSC primarily consists of macropores. These findings corroborate the TEM observations, indicating that CoSA/CoNP-NSDNC exhibits a hierarchical multiporous amorphous structure and emphasizes its larger specific surface area, which is consistent with the above TEM results.

The defects present in the samples were analyzed using Raman spectroscopy. As shown in Fig. 2d, the Raman spectra display peaks around 1350 and 1580 cm⁻^1^, representing the defect carbon (D-band) and graphitic carbon (G band), respectively [32, 33]. The intensity ratio of the D-band to the G band (*I*_D_/*I*_G_) is typically used to analyze the degree of defect of carbon. The *I*_D_/*I*_G_ value for CoSA/CoNP-NSDNC (1.05) is higher than that for Co-NSC (1.01), indicating a higher degree of defects in CoSA/CoNP-NSDNC. To further clarify the types of defects in CoSA/CoNP-NSDNC and Co-NSC, their Raman spectra were deconvoluted into D_4_ band (polyene) at 1200 cm⁻^1^, D_1_ band (graphene layer edges) at 1350 cm⁻^1^, D_3_ band (topological defects) at 1500 cm⁻^1^, G band (graphite lattice) at 1580 cm⁻^1^, and D_2_ band (surface graphene layers) at 1620 cm⁻^1^ [15]. Based on the fitting results in Fig. 2e and Table S1, CoSA/CoNP-NSDNC exhibits a higher proportion of the D_3_ band related to topological defects, while the percentages of the D_1_, D_2_, and G bands linked to graphitic structures are lower. These results indicate the presence of a greater number of pentagonal topological defects in CoSA/CoNP-NSDNC. The D_3_/G area ratio for CoSA/CoNP-NSDNC (1.11) is higher than that for Co-NSC (0.81), further confirming this result [32, 34].

The surface chemical states of the samples were characterized by XPS. The XPS survey spectra (Fig. S4) and the corresponding element contents analysis (Table S2) confirm the presence of C, N, S, and Co elements in the both samples. The high-resolution C 1*s* spectra for CoSA/CoNP-NSDNC and Co-NSC in Fig. 2f were deconvoluted into C-C/C=C (graphitic carbon) at 284.69 eV, C-S (carbon–sulfur bond) at 285.4 eV, C=N/C-O at 286.2 eV, and C-N at 289.3 eV [35, 36]. The corresponding content percentages in Table S3 indicate similar relative contents of each peak for CoSA/CoNP-NSDNC and Co-NSC, suggesting a similar surface chemical environment. Moreover, the N 1*s* spectra were deconvoluted in Fig. 2g into pyridinic N at 398.3 eV, Co-N_x_ at 399.1 eV, pyrrolic N at 400.1 eV, graphitic N at 401.35 eV, and oxidized N at 403.5 eV [20]. The fitting results in Table S4 indicate that CoSA/CoNP-NSDNC has higher contents of pyrrolic N and graphitic N than Co-NSC. Noteworthily, pyrrolic N is considered to conducive to the 2e⁻ ORR reaction, and higher graphitic N content suggests that Co-N_x_ species are embedded in the carbon matrix. The high-resolution S 2*p* spectra in Fig. 2h were deconvoluted into S 2*p*_3/2_ at 163.5 eV, S 2*p*_1/2_ at 165 eV, and sulfate at 167.6 eV [16]. The fitting results in Table S5 further confirm the incorporation of S elements in CoSA/CoNP-NSDNC and Co-NSC. The high-resolution Co 2*p* spectra in Fig. 2i were deconvoluted into Co⁰ at 778.2 eV, Co^2^⁺ at 779.2 and 794.6 eV, Co-N_x_ at 780.7 and 796.1 eV, and Co^3^⁺ at 782 and 797.4 eV [37, 38]. The fitting results (Table S6) indicate that in CoSA/CoNP-NSDNC and Co-NSC, a small portion of Co exists as Co nanoparticles embedded in the carbon framework, while most of the Co exists in the form of Co^2^⁺-N coordinated single atoms or as Co^3^⁺.

### Electrochemical Performances

**Fig. 3:**
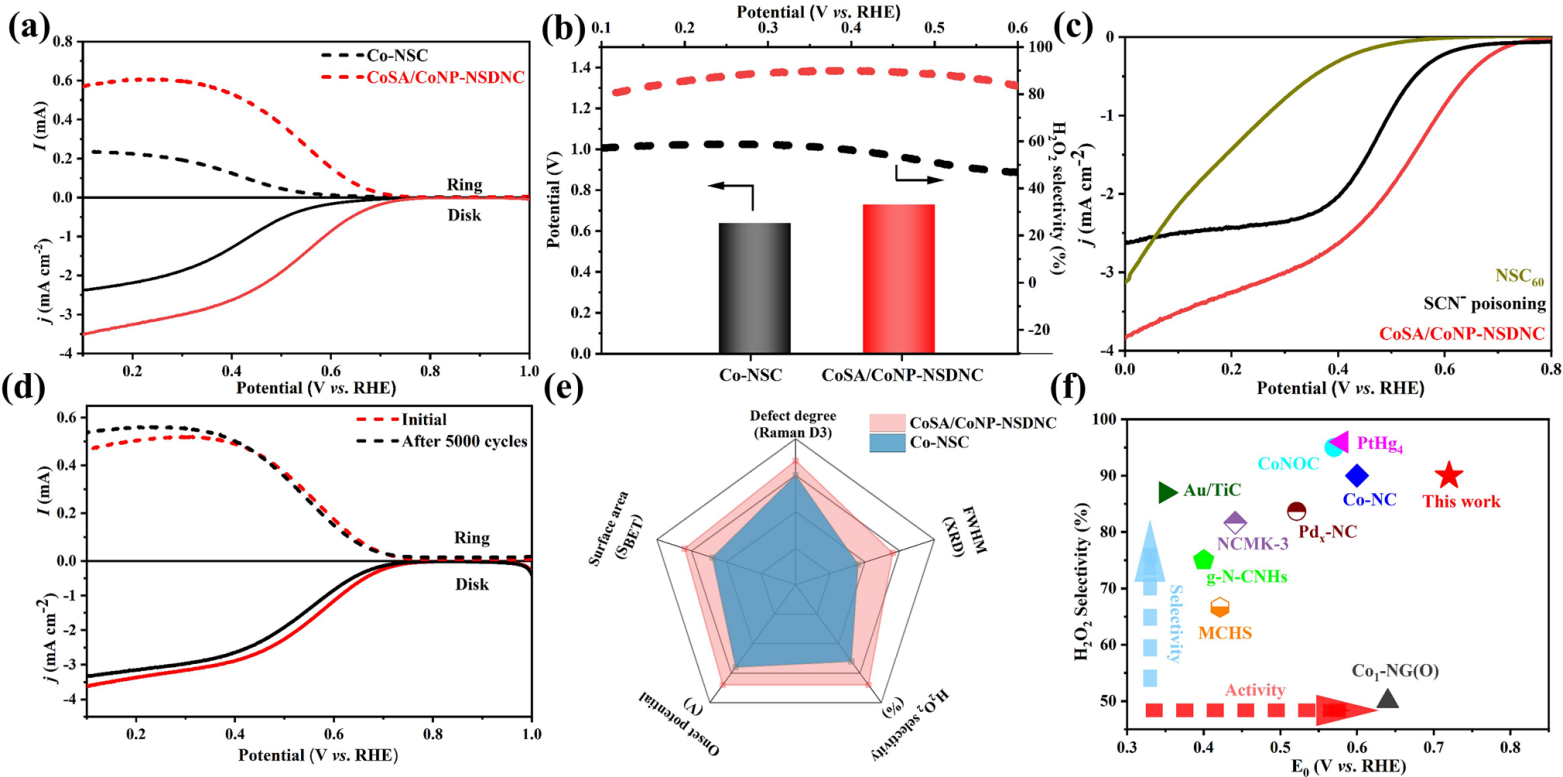
**Evaluation** **of electrochemical** **2e**^−^ **ORR** **performance of CoSA/CoNP-NSDNC and Co-NSC**. **a** Linear sweep voltammetry curves with disk and ring currents; **b** Corresponding calculated H_2_O_2_ selectivity and onset potential; **c** Polarization curves without cluster Co nanoparticles and Co single-atom sites; **d** CoSA/CoNP-NSDNC stability test linear sweep voltammetry curve; **e** CoSA/CoNP-NSDNC and Co-NSC comprehensive comparison chart. **f** Performance comparison of CoSA/CoNP-NSDNC with recently reported electrocatalysts

The ORR electrocatalytic performance of the samples was evaluated with a rotating ring-disk electrode (RRDE) in 0.1 M HClO_4_ solution within a three-electrode setup. During the tests, the rotation speed was maintained at 1600 rpm, and the Pt ring potential was held at 1.2 V (vs. RHE) to detect the H_2_O_2_ produced at the disk electrode. The linear sweep voltammetry (LSV) curves in Fig. 3a show that CoSA/CoNP-NSDNC has a higher disk current density and ring electrode current (detected H_2_O_2_ oxidation current) compared to Co-NSC, indicating higher ORR activity and H_2_O_2_ yield. Figure 3b compares the onset potential and H_2_O_2_ selectivity of CoSA/CoNP-NSDNC and Co-NSC catalysts. CoSA/CoNP-NSDNC demonstrates superior H_2_O_2_ selectivity, reaching up to 90%, over a broad potential range from 0.1 to 0.6 V. Furthermore, the onset potential of CoSA/CoNP-NSDNC is 0.72 V, significantly better than that of Co-NSC (0.64 V), demonstrating excellent electrochemical activity. These results indicate that the introduction of pentagonal topological defects in the carbon matrix can effectively enhance the ORR activity and H_2_O_2_ selectivity of the electrocatalyst. The active sites of the catalysts were further investigated using a thiocyanate ion (SCN⁻) poisoning experiment. SCN⁻ binds strongly to the locally coordinated single Co atom sites, selectively blocking the adsorption of reaction intermediates on these central metal active sites [24]. As shown in the polarization curve of CoSA/CoNP-NSDNC (Fig. S5), SCN⁻ poisoning of the coordinated Co atoms leads to a marked reduction in both onset potential and disk current. Additionally, in order to further verify the effect of Co nanoparticles on the catalytic performance, a NS-doped fullerene-based catalyst without Co nanoparticle clusters, NSC_60_, was synthesized. The comparative experiments (Fig. 3c) demonstrated that NSC_60_, which lacks Co nanoparticle clusters, exhibited a lower onset potential and current compared to both the SCN⁻-poisoned and pristine samples. Therefore, in addition to Co single atoms, the presence of stable Co nanoparticles also plays a positive role in the 2e⁻ ORR process in this study. Overall, these results confirm that the synergistic interaction between Co single atoms and Co nanoparticle clusters in CoSA/CoNP-NSDNC is crucial to its superior electrochemical performance.

Remarkably, the CoSA/CoNP-NSDNC catalyst demonstrates outstanding stability in acidic conditions. As shown in Fig. 3d, the LSV curve of the CoSA/CoNP-NSDNC catalyst was tested after continuous 5000 CV cycles, showing no decay compared to the initial curve. To further explore the electrocatalytic stability of CoSA/CoNP-NSDNC, a chronoamperometric test was conducted using the RRDE system. As shown in Fig. S6, the disk and ring currents showed slight attenuation after 10 h of testing, further indicating high electrochemical stability of CoSA/CoNP-NSDNC. Figure 3e presents a comprehensive comparison between CoSA/CoNP-NSDNC and Co-NSC. Compared to Co-NSC, CoSA/CoNP-NSDNC exhibits a higher specific surface area, more topological defects, and enhanced two-electron oxygen reduction performance. Noteworthy, compared with previously reported results (Fig. 3f and Table S7), CoSA/CoNP-NSDNC exhibits relatively better ORR performance. These results demonstrate that the CoSA/CoNP-NSDNC catalyst has outstanding ORR performance and stability in acidic environments, making it suitable for long-term electrochemical applications.

To understand the origin of the high catalytic performance of the CoSA/CoNP-NSDNC, the electrochemical active surface area (ECSA) and Tafel slope were analyzed. As shown in Figs. S7 and S8, CoSA/CoNP-NSDNC has a larger ECSA value than Co-NSC, indicating more available active sites. Tafel slope of CoSA/CoNP-NSDNC (105.2 mV dec^−1^) is lower than that of Co-NSC (200.18 mV dec^−1^), indicating faster H_2_O_2_ generation kinetics (Fig. S9). Overall, the outstanding H_2_O_2_ production performance of CoSA/CoNP-NSDNC is ascribed to its large specific surface area, hierarchical multiporous structure, abundant topological defects, and the synergistic effect between non-metallic single-atom doping and cluster metals. The extensive specific surface area and multiporous architecture enhance the exposure of active sites and expedite reactant transport [39–41]. The synergistic regulation of charge reconstruction between topological defects and non-metallic single atoms and cluster metals leads to a decrease in the band gap [42], thereby enhancing the catalytic performance of CoSA/CoNP-NSDNC.

To better understand the correlation between the structural aiders with the ORR performances, we further illustrate the impact of topological defects by screening the performances on nitrogen–sulfur–cobalt-doped carbon nanotubes (NSCoCNT), nitrogen–sulfur–cobalt-doped carbon black (NSCoXC), and nitrogen–sulfur–cobalt-doped porous carbon (NSCoPC) as counterparts. As shown in the XRD patterns in Fig. S10, compared to NSCoCNT, NSCoXC, and NSCoPC, CoSA/CoNP-NSDNC exhibits a broader (002) carbon crystal half-width at 26°, indicating a larger interlayer spacing and higher amorphousness. This observation suggests that the carbon matrix with pentagonal topological defects helps increase the defect level of carbon nanomaterials, thereby providing more active sites. The electrochemical performance in Fig. S11 strengthens that the C_60_ carbon matrix catalyst has larger ring currents, and higher H_2_O_2_ selectivity. To clarify the impact of heteroatoms on catalyst performance, nitrogen–sulfur-doped C_60_ (NSC_60_), nitrogen–cobalt-doped C_60_ (NCoC_60_), and sulfur–cobalt-doped C_60_ (SCoC_60_) were prepared. As shown in the electrochemical performance in Fig. S12, compared to NSC_60_, NCoC_60_, and SCoC_60_, CoSA/CoNP-NSDNC exhibits larger ring currents, higher H_2_O_2_ selectivity, and a more two-electron ORR reaction. These findings reveal that the exceptional electrochemical performance of the CoSA/CoNP-NSDNC catalyst is a result of the synergistic interplay among NSCo heteroatoms, clustered metal nanoparticles, and pentagonal topological defects.

**Fig. 4:**
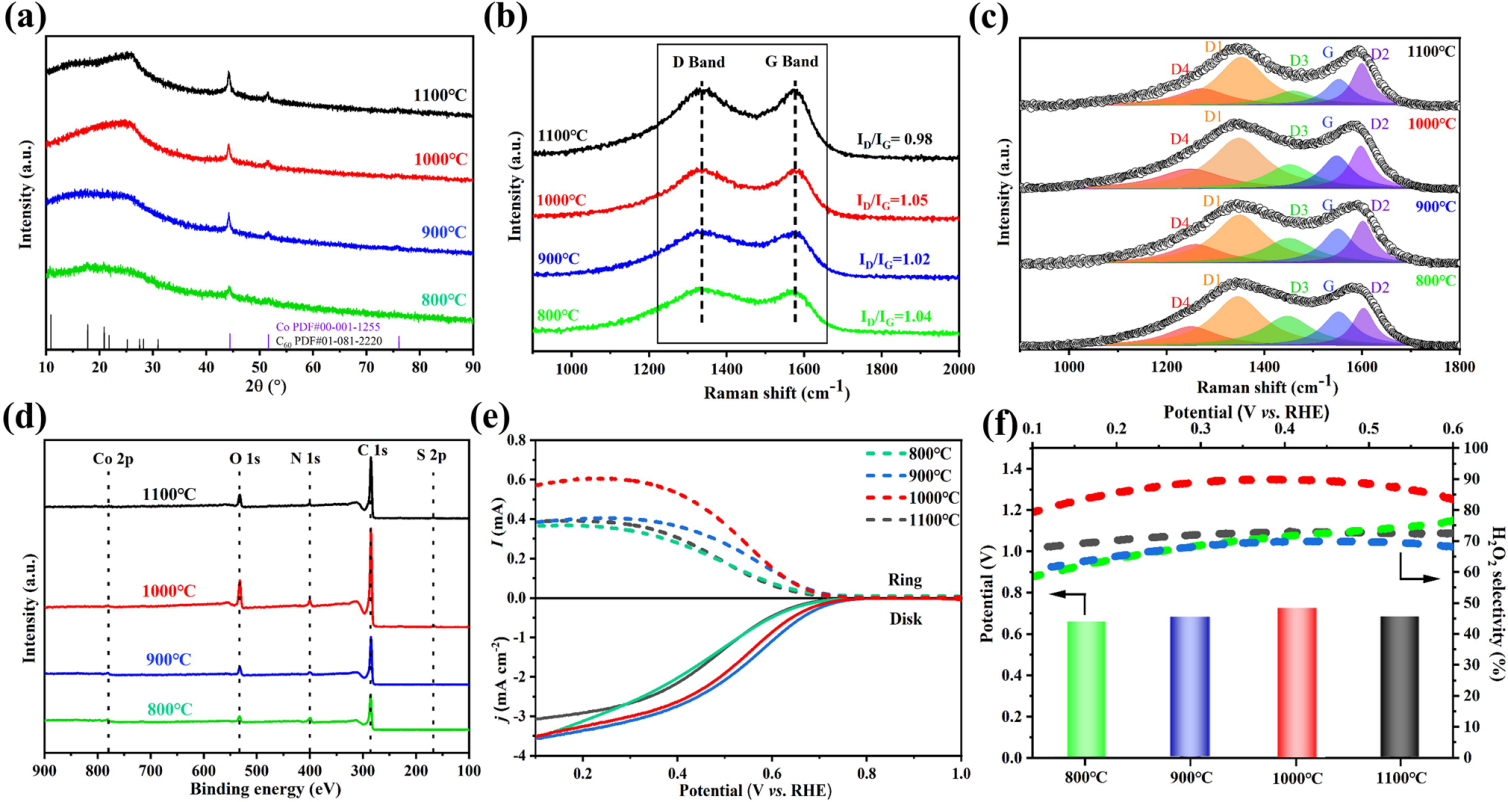
**Visualize the structural aiders in the performance boost. Oxygen reduction performance of CoSA/CoNP-NSDNC samples synthesized at (800–1100 °C)**. **a** XRD, **b, c** Raman, and **d** XPS survey of CoSA/CoNP-NSDNC samples synthesized at varying temperatures. **e** Linear sweep voltammetry (LSV) curves with disk and ring currents at a scan rate of 10 mV s^─1^. **f** Calculated H_2_O_2_ selectivity (solid line) and onset potential of the corresponding samples

In addition, the effect of pyrolysis temperature on the structural and catalytic properties of CoSA/CoNP-NSDNC materials was investigated. Figure 4a exhibits the XRD patterns of CoSA/CoNP-NSDNC-X (X = 800, 900, 1000, and 1100 °C) samples. Notably, when the pyrolysis temperature surpasses 900 °C, the materials display distinct peaks associated with noncrystalline carbon, suggesting a reorganization of the carbon structure. Additionally, compared to CoSA/CoNP-NSDNC-1100, CoSA/CoNP-NSDNC-1000 has a wider half-width, indicating a larger interlayer spacing and higher amorphousness. The Raman in Fig. 4b also indicates that CoSA/CoNP-NSDNC-1000 has the highest *I*_*D*_/*I*_*G*_ value (1.05), suggesting a higher degree of defects. The deconvolution of Raman spectra for samples at different temperatures (Fig. 4c and Table S8) shows that the percentage of topological defects associated with the D_3_ band gradually decreases with increasing temperature, reaching an optimal content for the two-electron ORR reaction at 1000 °C (Fig. 4e, f). The XPS results in Fig. 4d and Table S9 indicate that the pyrrolic N content is the highest in the CoSA/CoNP-NSDNC-1000 material at 1000 °C, which is conducive to 2e⁻ ORR. The electrocatalytic performance of CoSA/CoNP-NSDNC prepared at different temperatures is shown in Fig. 4e, f. The linear sweep voltammetry curves in Fig. 4e show that the detected H_2_O_2_ ring current first increases and then decreases with the increase in temperature, and CoSA/CoNP-NSDNC-1000 shows the best 2e⁻ ORR performance. Similarly, as shown in Fig. 4f, the initial potential (*E₀*) and H_2_O_2_ selectivity of samples also show a trend of first increasing and then decreasing, and they can reach the maximum value at 1000 °C. Therefore, CoSA/CoNP-NSDNC-1000 was chosen as the optimal catalyst. Additionally, the impact of raw material ratio on catalyst performance was studied. The electrochemical performance in Fig. S13a, b indicates that with increasing N and Co content, the ring current first increases and then decreases, among which the 10-1-0.1 CoSA/CoNP-NSDNC sample exhibiting the best electrochemical performance in H_2_O_2_ synthesis.

### H_2_O_2_ Generation and Applications

**Fig. 5:**
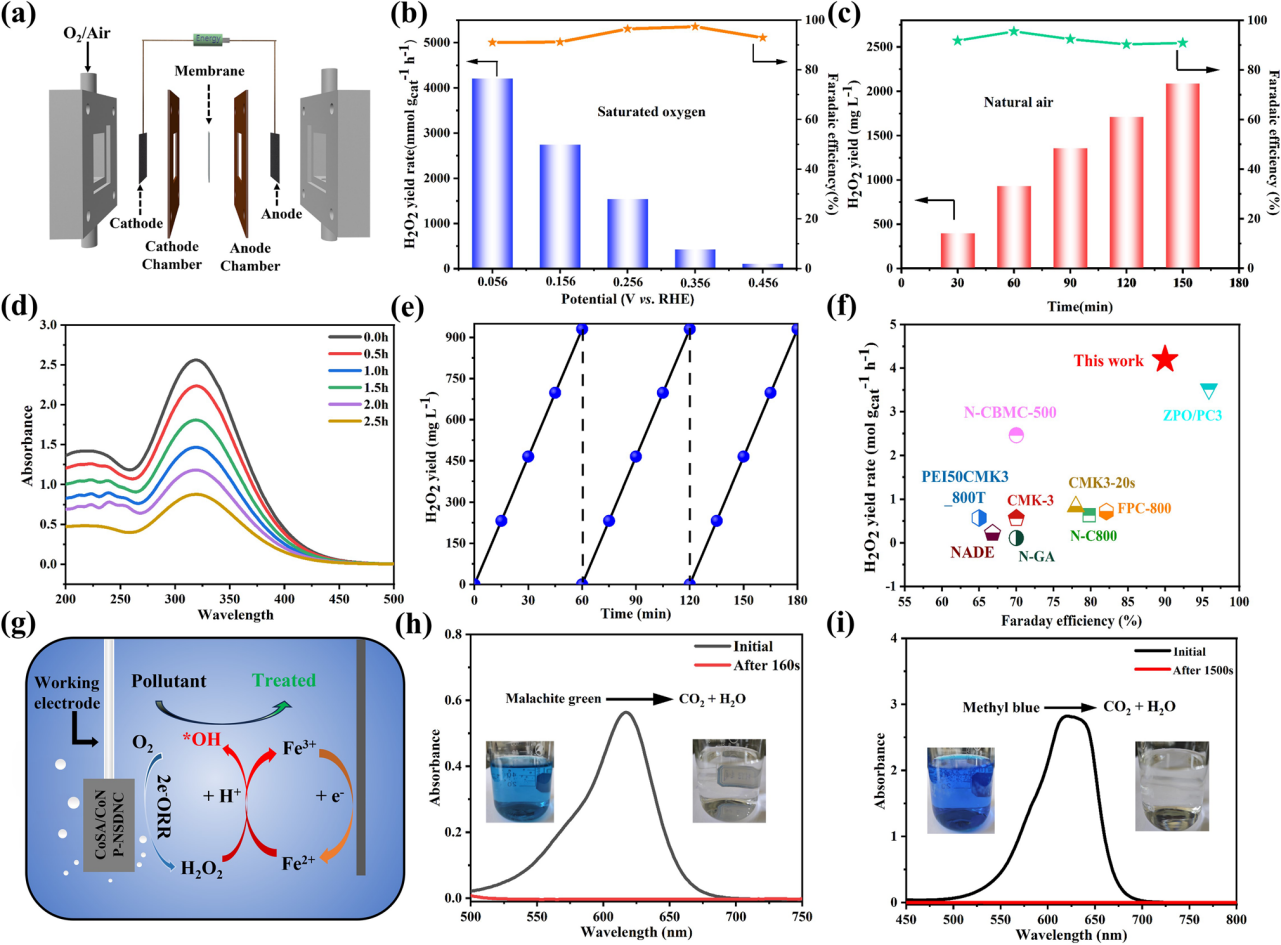
**Practical application performance of CoSA/CoNP-NSDNC**. **a** Schematic of the homemade flow cell setup; **b** H_2_O_2_ yield and Faraday efficiency across various potentials; **c** H_2_O_2_ yield and Faraday efficiency after 150 min of electrolysis at 0.056 V (vs. RHE) in ambient air; **d** UV–visible spectra of Ce^4+^ solution at varying concentrations; **e** Cyclic stability at 0.056 V (vs. RHE); **f** Comparison of H_2_O_2_ yield for CoSA/CoNP-NSDNC with other recent catalysts; **g** Schematic of degradation devices; absorbance curves and optical images of **h** methyl blue and **i** malachite green (50 mg L^−1^) before and after degradation in a three-electrode flow cell

To test the practical production capability of the CoSA/CoNP-NSDNC catalyst, a flow cell was assembled using CoSA/CoNP-NSDNC as the cathode catalyst (Fig. 5a). Under O_2_-saturated acidic conditions, CoSA/CoNP-NSDNC achieved a production rate of 4206.96 mmol g_cat_^-1^ h^-1^ at a potential of 0.056 V, with a Faradaic efficiency approaching 95% (Fig. 5b). Therefore, 0.056 V potential was selected for H_2_O_2_ accumulation test under natural environmental conditions. As shown in Fig. 5c, H_2_O_2_ accumulated to 2086.72 mg L⁻^1^ after 150 min, and the Faradaic efficiency remained stable. Figure 5d shows the absorbance of Ce(SO_4_)_2_ at different time intervals for cumulative H_2_O_2_ production. It can be observed that the absorbance decreases uniformly over time, indicating stable H_2_O_2_ production. In the cyclic stability tests (Fig. 5e), CoSA/CoNP-NSDNC also performed well, demonstrating its excellent stability. Figure 5f and Table S10 compare the production rate and Faradaic efficiency of recently reported 2e^−^ ORR electrocatalysts. Among them, the CoSA/CoNP-NSDNC exhibits excellent performance for H_2_O_2_ production. These results suggest that the CoSA/CoNP-NSDNC catalyst has excellent stability and strong application potential. Additionally, the catalytic Fenton degradation ability was tested in a three-electrode system (Fig. 5g). Using malachite green and methylene blue as substrates, the electrochemical degradation of organic substances at a concentration of 50 mg L^-1^ showed respectively rapid degradation performance within 3 and 25 min, demonstrating good degradation capability for organic pollutants (Fig. 5h, i). These findings demonstrate that the CoSA/CoNP-NSDNC catalyst possesses not only outstanding H_2_O_2_ production efficiency, but also a high capability for degrading organic pollutants.

## Conclusion

In summary, this work successfully synthesized a carbon-based 2e⁻ ORR electrocatalyst featuring the synergistic interaction of NSCo single-atom doping and Co nanoparticle clusters. The synthesized CoSA/CoNP-NSDNC possesses a high specific surface area, a hierarchical porous architecture, and abundant topological defects. This expansive surface area, combined with the hierarchical porosity, increases active site exposure and promotes more efficient reactant transport. The synergistic effects between heteroatoms, topological defects, and nanoparticle clusters modulate charge redistribution, which provides optimal binding strength for the adsorption and desorption of intermediates, thereby enhancing the 2e^− ^ORR electrocatalytic performance and stability of CoSA/CoNP-NSDNC. In practical applications, when CoSA/CoNP-NSDNC was employed in a flow cell, it achieved an excellent H_2_O_2_ production rate of 4206.96 mmol g_cat_⁻^1^ h⁻^1^, with a Faradaic efficiency approaching 95%. Furthermore, CoSA/CoNP-NSDNC demonstrated excellent organic pollutant degradation capability in the degradation of MG and MB via a Fenton-like reaction. This study on the synergistic effects between topological defects, non-metal or non-noble metal single-atom sites, and metal clusters provides valuable insights for the understanding and development of two-electron oxygen reduction electrocatalysts.

## Supplementary Information

Below is the link to the electronic supplementary material.

Supplementary file1 (DOCX 2797 KB)
