# Supplementary material for: Synergistic Single-Atom and Clustered Cobalt Sites on N/S Co-Doped Defect Nano-Carbon for Efficient H2O2 Electrosynthesis
Source: Nanomicro Lett. 2025 Feb 12;17:142. doi: 10.1007/s40820-025-01657-9 (PMC11822182; doi:10.1007/s40820-025-01657-9)
Supplement: Supplementary file 1 — Supplementary file1 (DOCX 2797 KB) [file 40820_2025_1657_MOESM1_ESM.docx]

Supporting Information for

**Synergistic Single-Atom and Clustered Cobalt Sites on N/S Co-Doped Defect Nano-Carbon for Efficient H_2_O_2_ E****lectrosynthesis**

Yuzhong Huang^1,#^, Chang Zhang^2,#^, Xingyu Wang^1,2,#^, Yuji Wu^3^, Jun Lv^1^, Jian Zhang^2^, Wangqiang Shen^1,4,*^ and Xing Lu^2,*^

^1^School of Materials Science and Engineering, Hefei University of Technology, Hefei 230009, P. R. China

^2^School of Materials Science and Engineering, Huazhong University of Science and Technology, Wuhan 430074, P. R. China

^3^School of Nuclear Engineering, Rocket Force University of Engineering, Xi’an 710025, P. R. China

^4^Engineering Research Center of High-Performance Copper Alloy Materials and Processing, Ministry of Education, Hefei University of Technology, Hefei 230009, P. R. China

^#^ Yuzhong Huang, Chang Zhang, and Xingyu Wang contributed equally to this work.

^*^Corresponding authors. E-mail: [lux@hust.edu.cn](mailto:lux@hust.edu.cn) (Xing Lu); [shenwq@hfut.edu.cn](mailto:shenwq@hfut.edu.cn) (Wangqiang Shen)

# S1 Chemicals and materials

Fullerene, melamine, L-cysteine, cobalt nitrate hexahydrate, perchloric acid, ethanol, isopropanol, sulfuric acid, ferric sulfate heptahydrate, cerium sulfate, NaSCN, malachite green, methyl blue, and Nafion (5 wt. %) are all from Sigma. Odrich Reagent Co., Ltd. Carbon fiber paper (CFP) was purchased from Toray Plastic Precision Co., Ltd. Co., Ltd. Commercial titanium-based IrO_2_ coated electrode was purchased from Siotech Industrial Technology Co., Ltd. All chemical reagents are directly used in the experiment without further purification.

# S2 Experimental section

Dissolve 0.1g of cobalt nitrate hexahydrate was dissolved in 20 mL of pure water to obtain a cobalt nitrate hexahydrate solution for further use. 0.2g of fullerene was dissolved in 20 mL of CS_2_, and then 4g of melamine and 0.4g of L-cysteine were mixed, grounded, and poured into CS_2_ containing fullerene at 200 rpm. The mixture was heated in a water bath at 60 °C until CS_2_ was completely volatilized, and then cobalt nitrate hexahydrate solution was added. After ultrasonication for 20 min, the mixture was dried at 70 °C and ground to obtain a mixture precursor. The mixture precursor obtained in 2.1 was heated in an argon atmosphere in two stages. The first stage was heated to 600 °C and held for 1h. The second stage was heated to 800-1100 °C and held for 2h (heating rate was 5 °C per minute). Subsequently, the acid leaching method was used to remove the inactive Co material in the framework by heating in 0.5 M H_2_SO_4_ solution at 60 °C for 8 h. The samples were collected by suction filtration, washed to pH = 7, and dried overnight under vacuum conditions to obtain a black powder, defined as CoSA/CoNP-NSDNC. Similarly, except for the removal of fullerene in 2.1 and the second stage of 2.2 at 1000 °C for 2h, the other conditions remain unchanged, and the product is defined as Co-NSC. For comparison, the mixture precursor was annealed at 700-1000 °C in an argon atmosphere, and the prepared product was defined as CoSA/CoNP-NSDNC-X (X = 700-1100 °C).

# S3 Structural characterizations

The SEM images were captured using the SU8020 equipment. The TEM visualizations were acquired with the JEM-2100F microscope. XRD analyses of the synthesized materials were conducted on an X'Pert PRO MPD diffractometer, scanning from 10° to 90° using Cu-Kα radiation (λ=1.5418 Å). The Raman spectra were recorded with a LabRAM HR800 device at 532 nm. XPS analyses were performed on the AXIS-ULTRA DLD-600W system. Nitrogen adsorption and desorption profiles were studied at 77 K using the ASAP 2460 instrument from Micromeritics, USA. The Brunauer-Emmett-Teller (BET) method and the density functional theory (DFT) were employed to calculate specific surface areas and pore size distribution, respectively. UV-vis spectroscopic analyses were done using the UV2600 Shimadzu apparatus.

# S4 Electrochemical measurements

The electrochemical tests were carried out using the conventional rotating ring-disk technique on a CHI 760E workstation at ambient temperature, employing an MSR Electrode Rotator from Pine Research Instrumentation. A rotating ring-disk electrode (RRDE) was utilized as a working electrode, with the gassy carbon disk electrode (area: 0.2475 cm^–2^) and the Pt ring electrode (area: 0.1866 cm^–2^). A graphite rod acted as the counter electrode, while a reversible hydrogen electrode (RHE) served as the reference electrode. For the catalyst ink formulation, 5 mg of catalysts was dispersed in a 1 mL solution mix of isopropyl alcohol and 0.1 wt % Nafion. After undergoing ultrasonic dispersion for 30 minutes, 5 μL ink was coated to the disk electrode, resulting in a catalyst loading of 0.1 mg cm^–2^. Linear sweep voltammetry (LSV) tests took place in an O_2_-saturated 0.1 M HClO_4_ electrolyte with a rotation speed of 1600 rpm and a scanning rate of 10 mV s^–1^. The ring electrode potential was set at 1.2 V to detect the produced H_2_O_2_. The electron-transfer number (*n*) and H_2_O_2_ selectivity (H_2_O_2_%) for the catalysts were determined using the following equations [S1]:

$\text{n}\text{= }\frac{\text{4×|}\text{I}_{\text{D}}\text{|}}{\text{|}\text{I}_{\text{D}}\text{|+}\frac{\text{I}_{\text{R}}}{\text{N}}}$ (S1)

$\text{H}_{\text{2}}\text{O}_{\text{2}}\text{\%}\text{ }\text{= }\frac{\text{200×}\frac{\text{I}_{\text{R}}}{\text{N}}}{{\text{|}\text{I}}_{\text{D}}\text{|+}\frac{\text{I}_{\text{R}}}{\text{N}}}$ (S2)

Where *I_D_* is the disk current, *I_R_* is the ring current, and *N* is the collection efficiency (0.37).

The H_2_O_2_ electroproduction kinetics were conducted based on the Koutecky-Levich equation:

$\frac{\text{1}}{\text{j}}\text{= }\frac{\text{1}}{\text{j}_{\text{K, }\text{H}_{\text{2}}\text{O}_{\text{2}}}}\text{+}\frac{\text{1}}{\text{j}_{\text{L, }\text{H}_{\text{2}}\text{O}_{\text{2}}}}$ (S3)

Tafel slopes were obtained based on the following equation:

$\text{η}\text{ }\text{=}\text{ }\text{a}\text{+}\text{b}\log\text{(}\text{ }\text{j}_{\text{K, }\text{H}_{\text{2}}\text{O}_{\text{2}}}\text{)}$ (S4)

Where $\text{j}_{\text{K, }\text{H}_{\text{2}}\text{O}_{\text{2}}}$ is the kinetic current density, $\text{j}_{\text{L, }\text{H}_{\text{2}}\text{O}_{\text{2}}}$ is the theoretical limiting current density of H_2_O_2_ (2.9 mA cm^–2^) at the rotation rate of 1600 rpm [S2], *η* is the overpotential, and *b* is the Tafel slope.

The electrochemical active surface area (ECSA) was calculated as the following equation:

$\text{C}_{\text{DL}}\text{= }\frac{\text{j}_{\text{c}}}{\text{v}}$ (S5)

Where *C_DL_* is the double-layer capacitance, $\text{j}_{\text{c}}$ is the current density of the double-layer capacitor, and $\text{v}$ is the scan rate.

# S5 H_2_O_2_ yield measurements

The H_2_O_2_ production processes were performed in the self-assembled flow cell with a three-electrode system [S3]. For the flow cell, a carbon paper with a microporous layer (2 × 2 cm) was used as the working electrode. An Ag/AgCl and a RuO_x_/Ti plate (2 × 2 cm) were applied as the reference electrode and counter electrode, respectively. The concentrations of H_2_O_2_ were measured using the traditional cerium sulfate Ce(SO_4_)_2_ titration technique. This approach is grounded on the principle that H_2_O_2_ turns the yellow Ce^4+^ solution into a colorless Ce^3+^ solution, as shown in the equation below. The consumed concentration of Ce^4+^ was measured photometrically at a wavelength of 319 nm using UV-vis spectroscopy.

$\text{2}\text{Ce}^{\text{4+}}\text{+}\text{H}_{\text{2}}\text{O}_{\text{2}}\text{→2}\text{Ce}^{\text{3+}}\text{+2}\text{H}^{\text{+}}\text{+}\text{O}_{\text{2}}$ (S6)

By linear fitting the absorbance values of a series of known concentrations of 0.01, 0.02, 0.05, 0.1, 0.2, 0.3, 0.4, and 0.5 mmol L^−1^ solution of Ce^4+^, the calibration curve was plotted [S4]. To prepare the 0.5 mmol L^−1^ Ce^4+^ standard solutions, 16.65 mg of Ce(SO_4_)_2_ were dissolved in 100 mL of 0.5 mol L^−1^ H_2_SO_4_. The sample solution was mixed with the standard Ce^4+^ solution by a volume ratio of 1: 100 to quantify the produced H_2_O_2_. The yield rate (YR) and Faraday efficiency (FE) for H_2_O_2_ generation were calculated as follows:

$\text{c}_{\text{H}_{\text{2}}\text{O}_{\text{2}}}\text{ }\left( \text{mM} \right)\text{= }\frac{\text{V}_{\text{Ce}^{\text{4+}}}\text{×}\text{c}_{\text{Ce}^{\text{4+}}} - {\text{(}\text{V}}_{\text{Ce}^{\text{4+}}} + \text{V}_{\text{ removed electrolyte}}\text{)}\text{ }\text{×}\text{ }{\text{c}_{\text{after}}}_{\text{Ce}^{\text{4+}}}}{\text{2}\text{ }\text{×}\text{ }\text{V}_{\text{removed electrolyte}}}$ (S7)

$\text{H}_{\text{2}}\text{O}_{\text{2}}\text{ production rate }\text{= }\frac{\text{c}_{\text{H}_{\text{2}}\text{O}_{\text{2}}}\text{ }\text{×}\text{ }\text{34}}{\text{t}}$ (S8)

$\text{FE}\left( \text{\%} \right)\text{ = }\frac{\text{c}_{\text{H}_{\text{2}}\text{O}_{\text{2}}}\text{ }\text{×}{\text{ }\text{V}}_{\text{electrolyte}}\text{ }\text{×}\text{ }\text{2}\text{ }\text{×}\text{ }\text{96,485}}{\text{∫}_{\text{0}}^{\text{t}}\text{i}\text{ }\text{dt}}$ (S9)

Where $\text{c}_{\text{H}_{\text{2}}\text{O}_{\text{2}}}$ is the molarity of generated H_2_O_2_, ${\text{c}_{\text{before}}}_{{\text{ }\text{Ce}}^{\text{4+}}}$ is the molarity of the Ce^4^*^+^* standard solution before the test, ${\text{c}_{\text{after}}}_{\text{Ce}^{\text{4+}}}$ is the molarity of the Ce^4+^ solution after the test, $\text{V}_{\text{Ce}^{\text{4+}}}$ is the volume of the Ce^4+^ standard solution, $\text{V}_{\text{removed electrolyte}}$ is the volume of the removed electrolyte under test, $\text{V}_{\text{electrolyte}}$ is the total volume of the electrolyte under test, and *t* is the time of duration.

# S6 Degradation by an electro-Fenton-like process

The degradation of organics (50 mg L^−1^ malachite green and methyl blue) was performed in the above three-phase flow cell by Electro-Fenton-like reactions. The degradation of Methylene Blue (MB) and Malachite Green (MG) through Fenton-like reactions involves the catalytic decomposition of hydrogen peroxide (H_2_O_2_) by transition metal catalysts (e.g., Fe) to generate highly reactive oxygen species (ROS), primarily hydroxyl radicals (•OH) (Fe^2+^ + H_2_O_2_ → Fe^3+^ + ·OH + OH^−^). These radicals exhibit strong oxidative ability, attacking the chromophores (e.g., C=N, C–S bonds) and aromatic rings of the dye molecules, leading to their structural breakdown and decolorization. Intermediate products are further oxidized into smaller, non-toxic molecules such as CO_2_ and H_2_O. Here, both the cathode and the anode chamber were filled with the same O_2_-saturated electrolyte (30 mL of PH=1 HClO_4_ solution contained 0.1 mmol Na_2_SO_4_, 0.5 mmol FeSO_4_·7H_2_O and 50 mg L^−1^ malachite green and methyl blue). The electrolysis current was set to 30 mA until the organics were completely degraded the solution became colorless, and the absorbance of the electrolyte in the cathode compartment was tested to determine the concentration of the organics.

**Supplementary Figures and Tables**


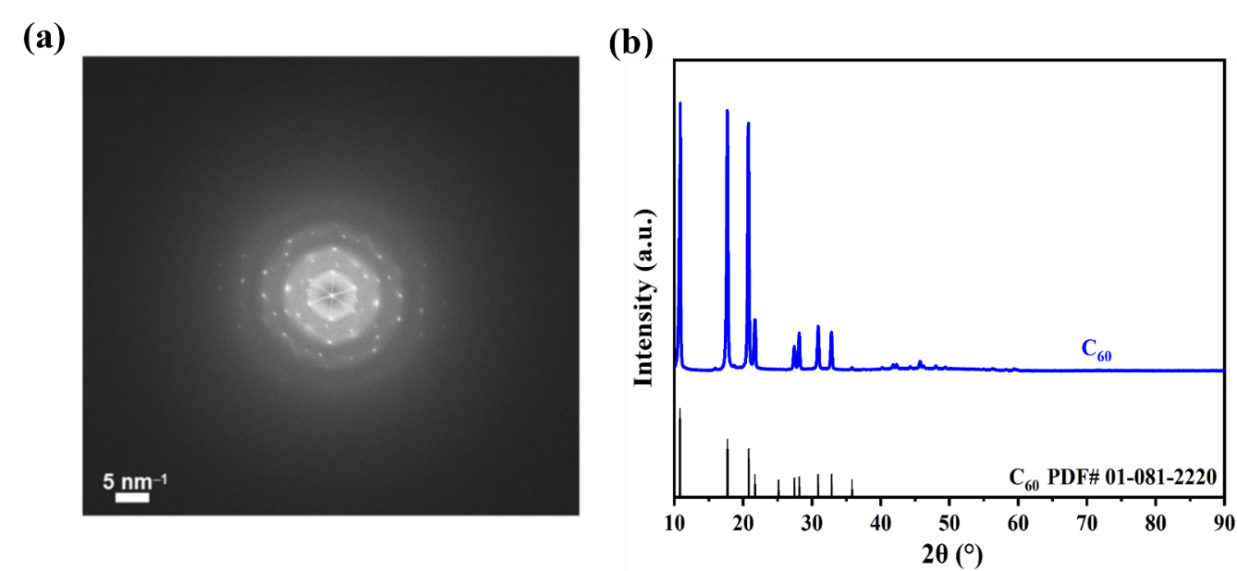


Fig. S1 Structure of the raw material. (**a**) SAED image, and (**b**) XRD pattern of C_60_


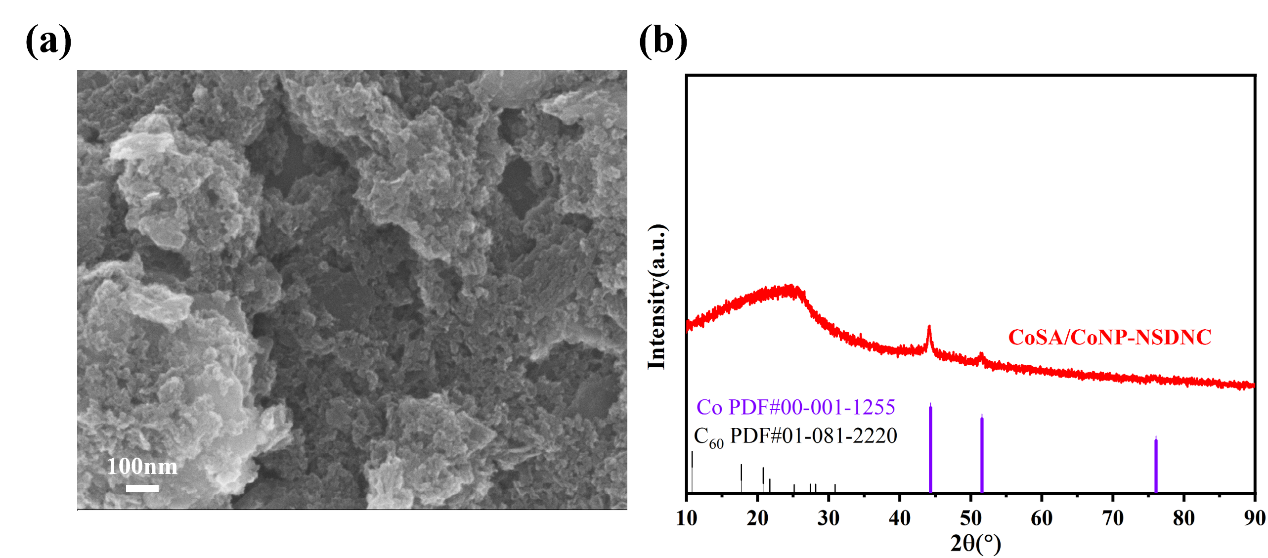


Fig. S2 (**a**) Scanning electron microscope and (**b**) XRD pattern of CoSA/CoNP-NSDNC


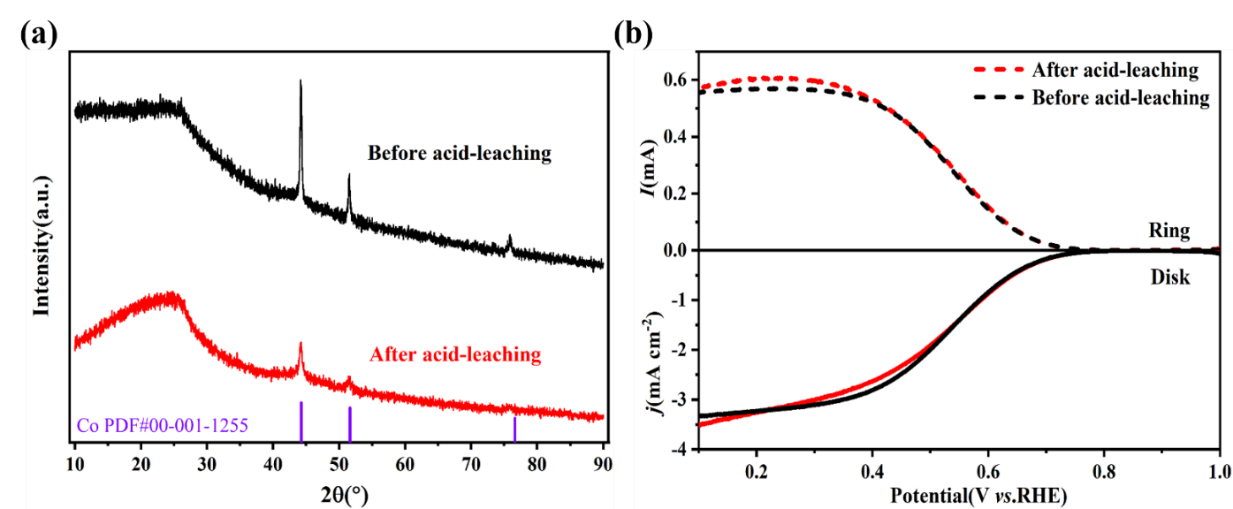


Fig. S3 (**a**) XRD patterns and (**b**) LSV curves of CoSA/CoNP-NSDNC before and after the acid-leaching process


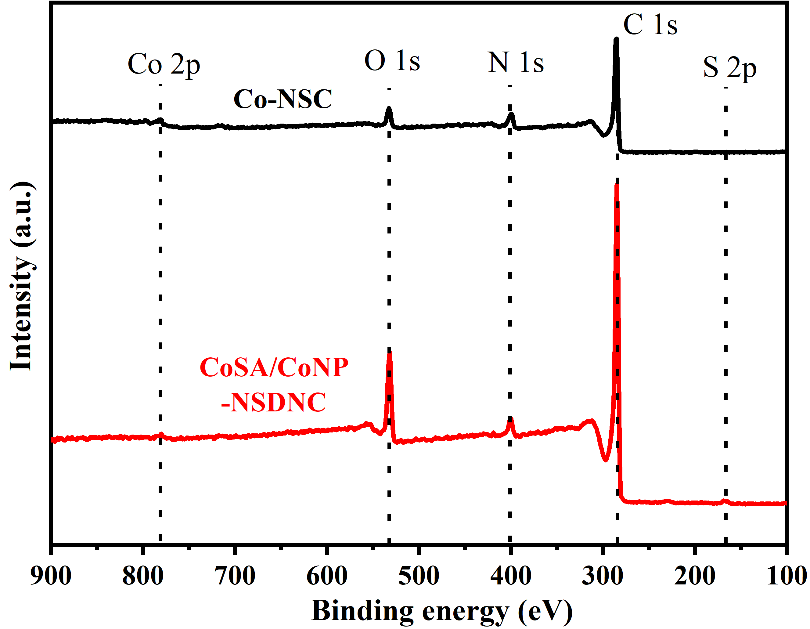


Fig. S4 Survey XPS spectra of CoSA/CoNP-NSDNC and Co-NSC


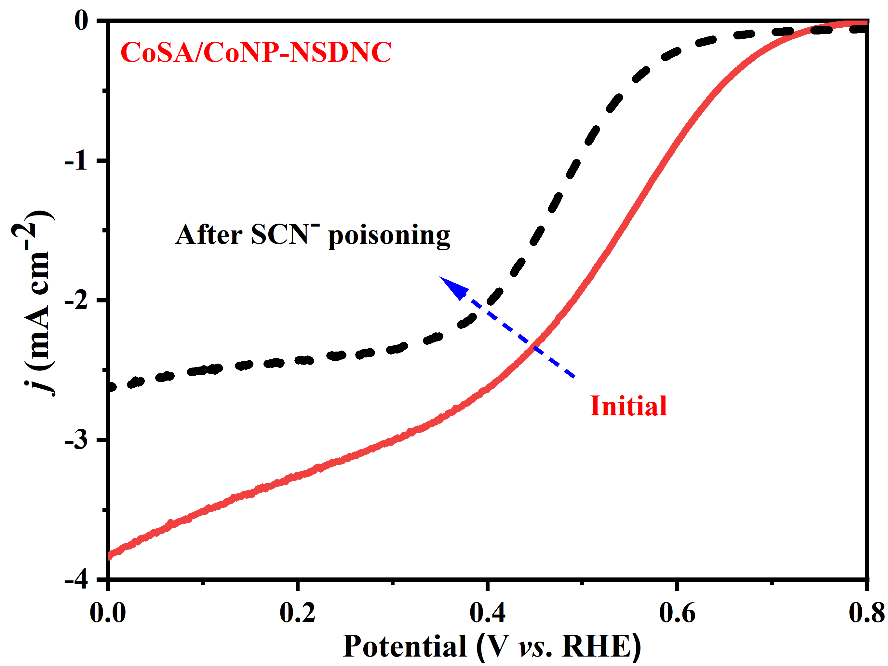


Fig. S5 LSV curves of CoSA/CoNP-NSDNC before and after the addition of 10 mM SCN^-^ in O_2_-saturated (pH=1) HClO_4_ electrolyte


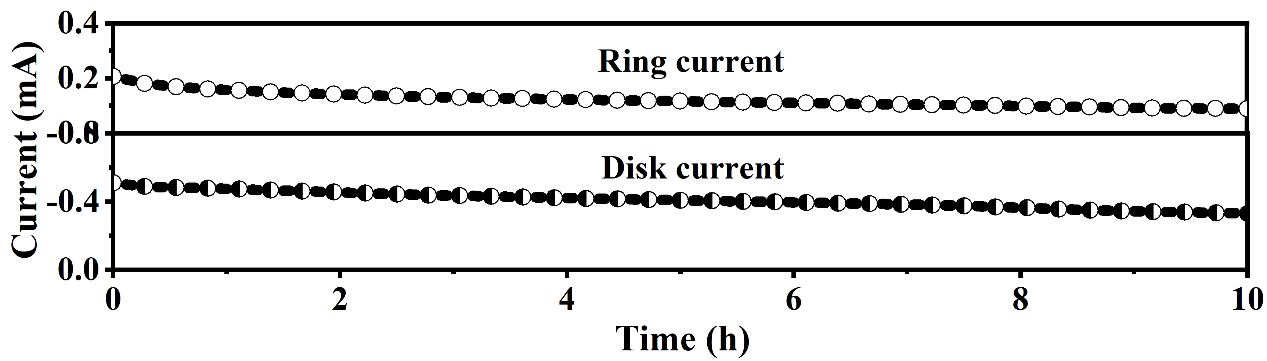


Fig. S6 Chronoamperometric oxygen-saturated (pH=1) HClO_4_ solution at disk potential of 0.3 V and ring potential of 1.2 V


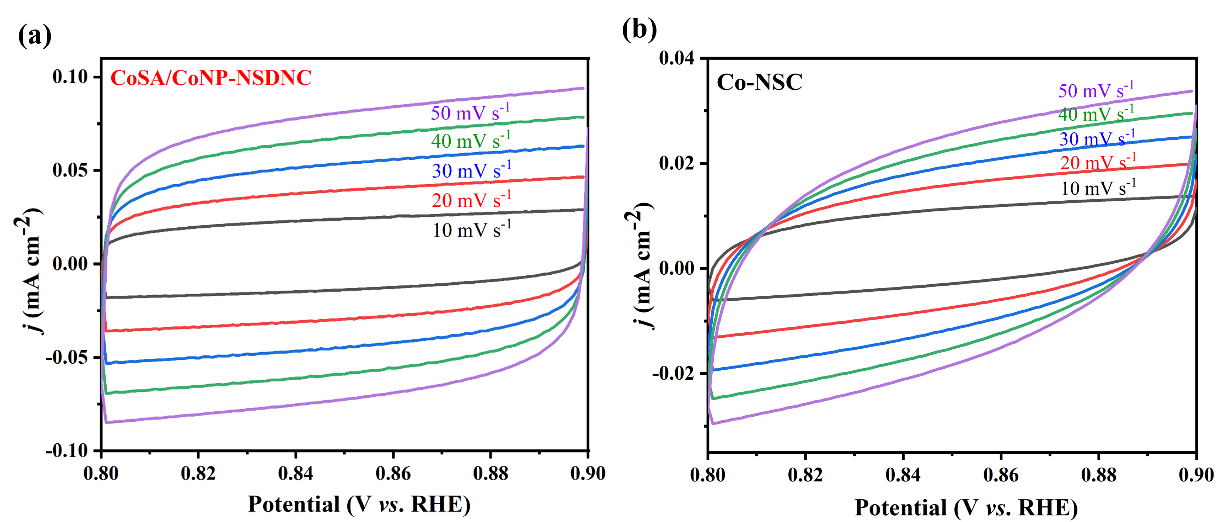


Fig. S7 CV curves of (**a**) CoSA/CoNP-NSDNC and (**b**) Co-NSC at scan rates of 10, 20, 30, 40, and 50 mV s^-1^ in nitrogen-saturated (pH=1) HClO_4_


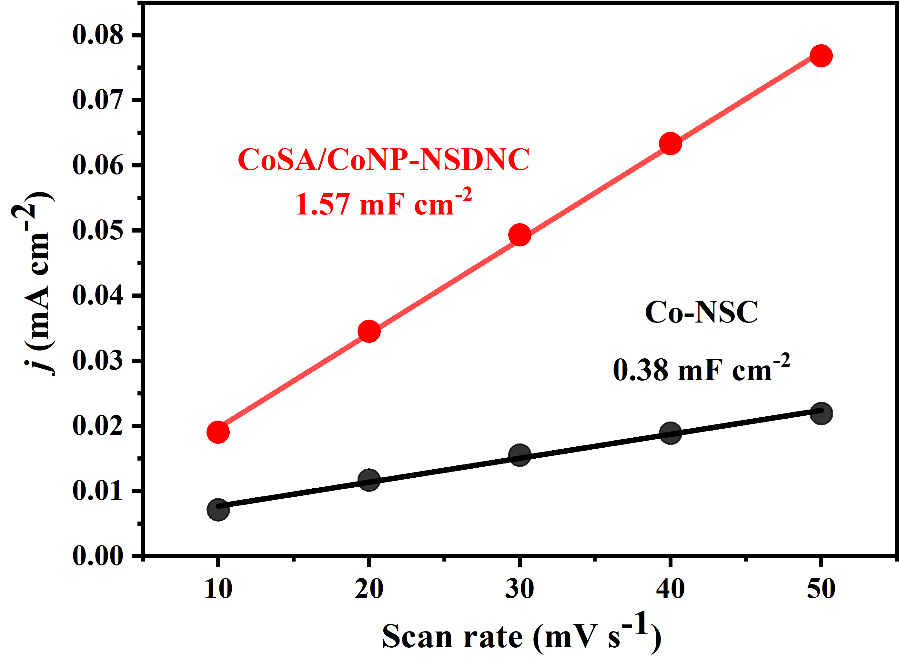


Fig. S8 Calculated *C_DL_* values of CoSA/CoNP-NSDNC and Co-NSC at the potential of 0.85 V


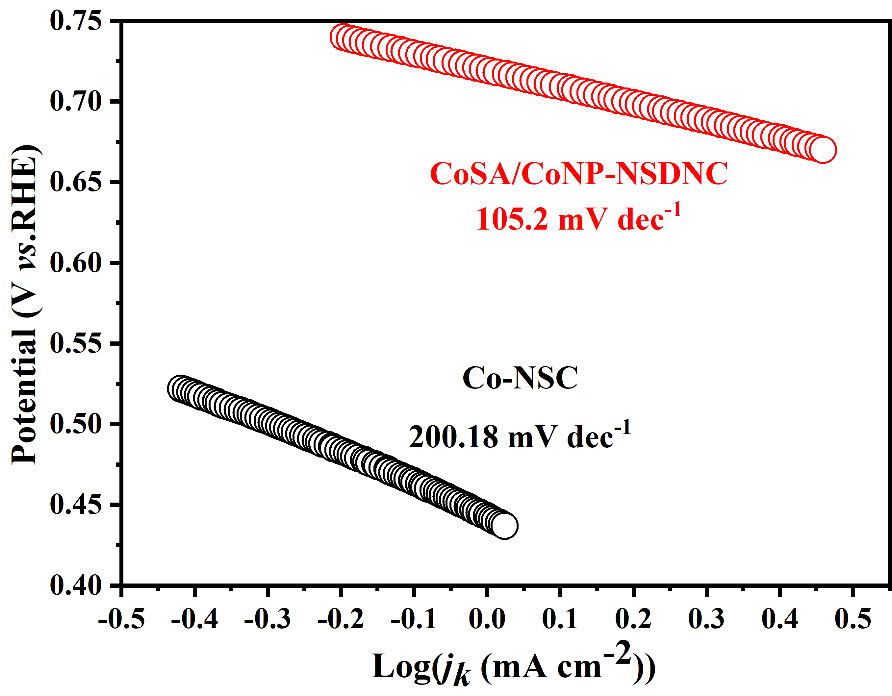


Fig. S9 Tafel plots extracted from the detective H_2_O_2_ currents of CoSA/CoNP-NSDNC and Co-NSC


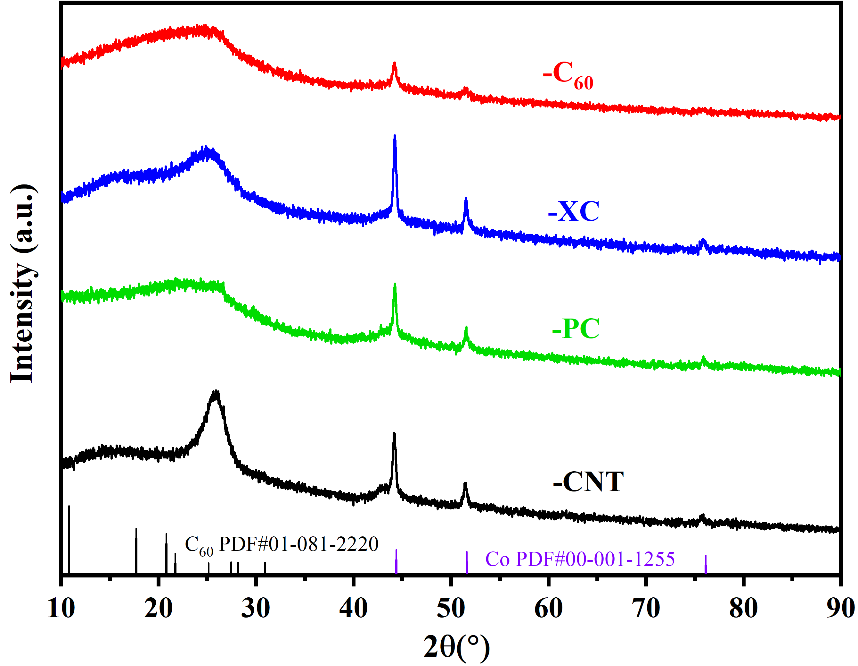


Fig. S10 XRD spectra of N, S, and Co doped with different carbon substrates


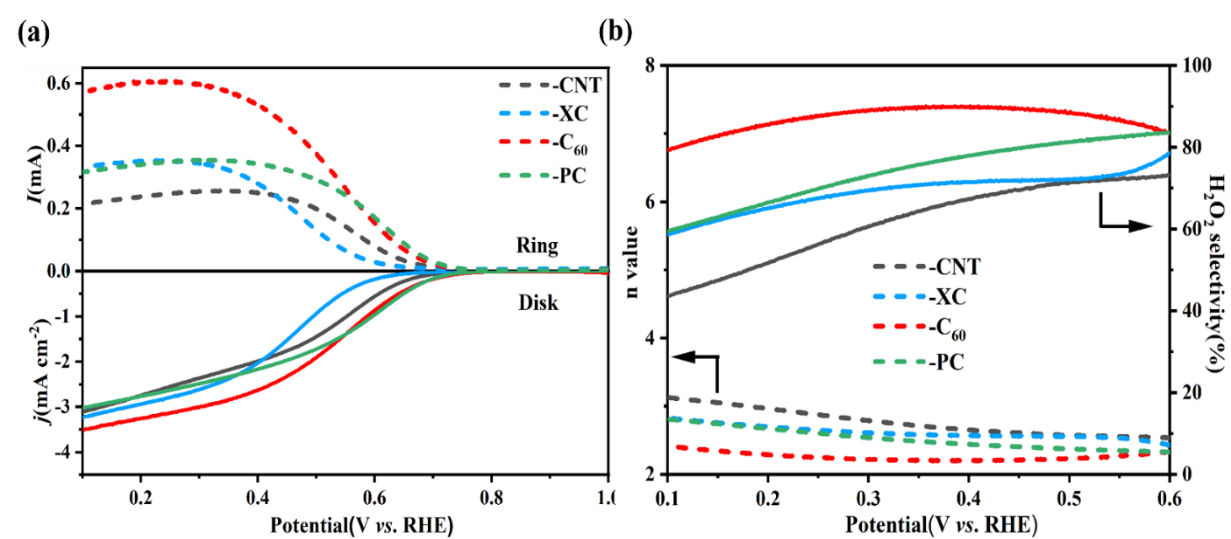


Fig. S11 Electrochemical properties of N, S and Co doped with different carbon substrates. (**a**) LSV curves with disk (solid line) and ring (dotted line) currents at a scan rate of 10 mV s^-1^. (**b**) corresponding calculated H_2_O_2_ selectivity (solid line) and electron transfer number n (dotted line)


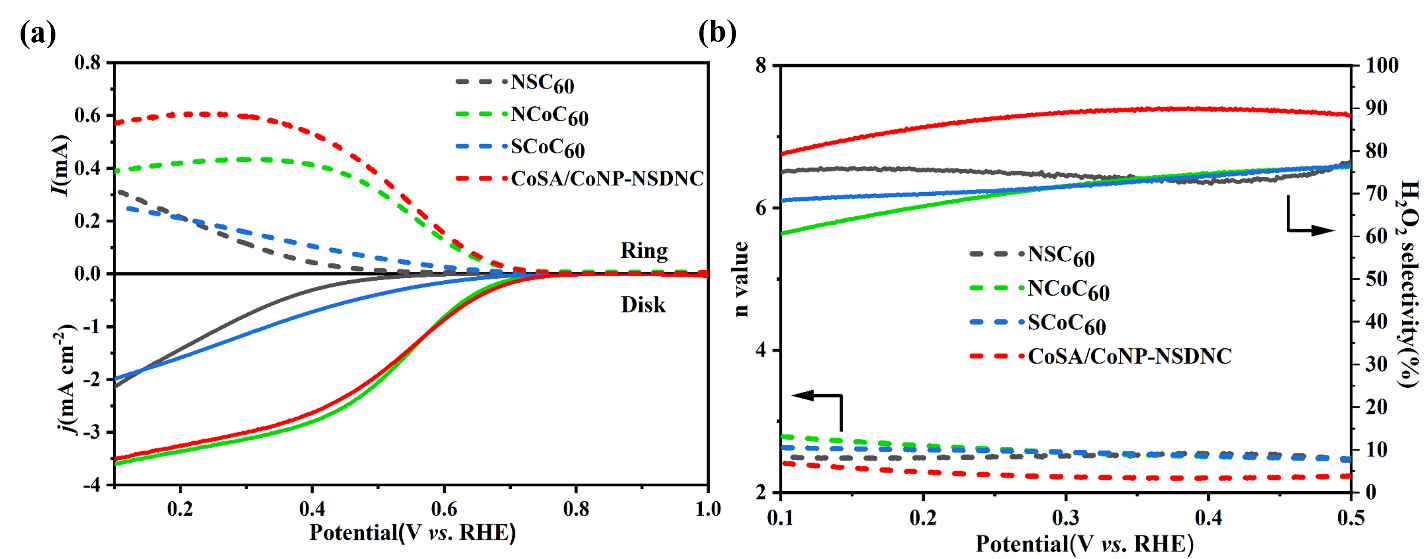


Fig. S12 Electrochemical properties of Cross double doped fullerene. (**a**) LSV curves with disk and ring currents at a scan rate of 10 mV s^-1^. (**b**) Corresponding calculated H_2_O_2_ selectivity (solid line) and electron transfer number n (dotted line)


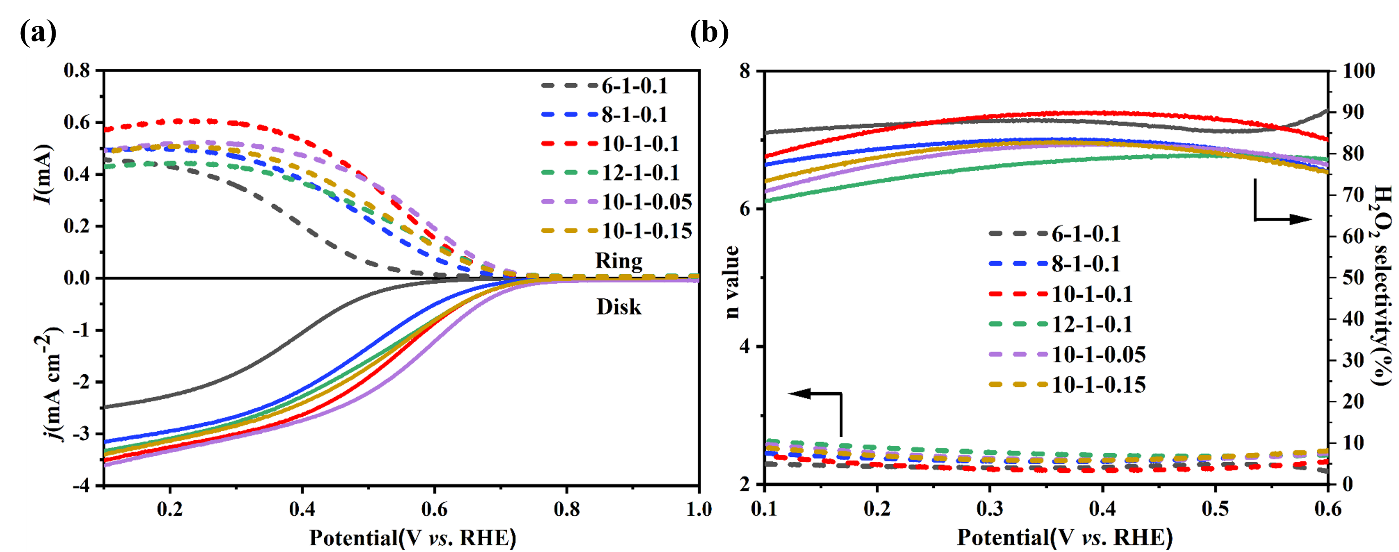


# Fig. S13 Electrochemical properties of CoSA/CoNP-NSDNC with different raw material ratios. (a) LSV curves with disk (solid line) and ring (dotted line) currents at a scan rate of 10 mV s^-1^. (b) Corresponding calculated H_2_O_2_ selectivity (solid line) and electron transfer number n (dotted line)


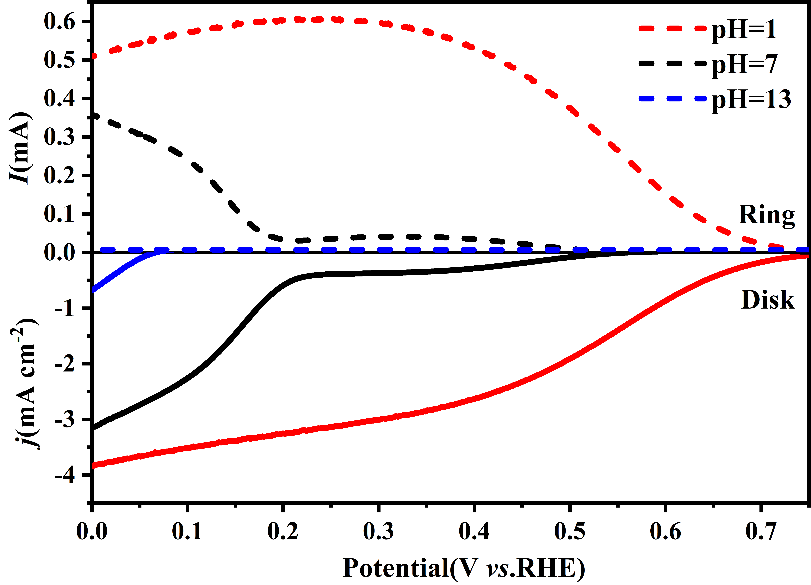


# Fig. S14 The linear sweep voltammetry curves of CoSA/CoNP-NSDNC samples in acidic, neutral and alkaline solutions

The **Fig. S14** above shows the linear sweep voltammetry curves of the CoSA/CoNP-NSDNC samples in electrolytes with different pH values. In the acidic electrolyte, the disk current density reaches 4 mA/cm², and the maximum ring current reaches 0.6 mA, demonstrating excellent electrochemical performance for oxygen reduction. In the neutral electrolyte, the disk current density reaches 3 mA/cm², and the maximum ring current is 0.35 mA. In the alkaline electrolyte, the ring current is almost 0 mA, indicating that no hydrogen peroxide is generated. The results shown in the Figure above suggest that the prepared CoSA/CoNP-NSDNC samples have the best electrochemical performance for oxygen reduction in the acidic electrolyte.


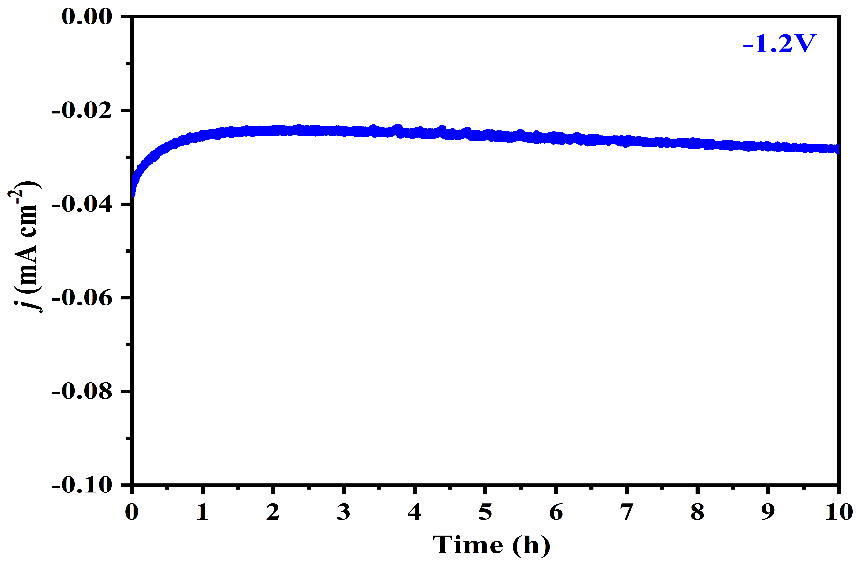


# Fig. S15 *i*-*t* curve of the CoSA/CoNP-NSDNC sample in the acidic electrolyte at -1.2 V

**Figure S15** above shows the *i-t* curve of CoSA/CoNP-NSDNC under acidic conditions. When operating under a constant voltage of -1.2 V for 10 hours, the current density of CoSA/CoNP-NSDNC has always remained above 10 mA/cm², demonstrating good stability under a relatively high current

Table S1 Raman characteristic properties of CoSA/CoNP-NSDNC and Co-NSC

| Catalyst | Intensity ratio of D and G | Area percentage of the D1 band (%) | Area percentage of the D2 band (%) | Area percentage of the D3 band (%) | Area percentage of the D4 band (%) | Area percentage of the G band (%) | Area ratio of D3 and G |
| --- | --- | --- | --- | --- | --- | --- | --- |
| Co-NSC | 1.01 | 38.52 | 15.01 | 14.08 | 15.17 | 17.22 | 0.81 |
| CoSA/CoNP-NSDNC | 1.05 | 38.29 | 13.66 | 15.85 | 17.92 | 14.28 | 1.11 |

Table S2 Element contents properties of CoSA/CoNP-NSDNC and Co-NSC

| Sample | C 1s（at%） | N 1s（at%） | O 1s（at%） | S 2p（at%） | Co 2p（at%） |
| --- | --- | --- | --- | --- | --- |
| Co-NSC | 82.21 | 11.2 | 5.01 | 0.59 | 0.98 |
| CoSA/CoNP-NSDNC | 85.38 | 3.96 | 10.17 | 0.24 | 0.24 |

Table S3 Content percentages from the decomposed C 1s spectra of CoSA/CoNP-NSDNC and Co-NSC

| Sample | C-C/C=C | C-S | C=N/C-O | C-N |
| --- | --- | --- | --- | --- |
| Co-NSC | 51.31 | 18.12 | 21.04 | 9.54 |
| CoSA/CoNP-NSDNC | 54.05 | 19.14 | 19.08 | 7.73 |

Table S4 Content percentages from the decomposed N 1s spectra of CoSA/CoNP-NSDNC and Co-NSC

| Sample | Pyridinic-N | Co-N_x_ | Pyrrolic-N | Graphitic-N | Oxide-N |
| --- | --- | --- | --- | --- | --- |
| Co-NSC | 9.28 | 25.38 | 32.1 | 28.66 | 4.57 |
| CoSA/CoNP-NSDNC | 8.94 | 8.16 | 39.59 | 29.1 | 14.21 |

Table S5 Content percentages from the decomposed S 2p spectra of CoSA/CoNP-NSDNC and Co-NSC

| Sample | S 2p^2/3^ | S 2p^1/2^ | Sulfate |
| --- | --- | --- | --- |
| Co-NSC | 31.47 | 15.74 | 52.79 |
| CoSA/CoNP-NSDNC | 38.95 | 19.48 | 41.57 |

Table S6 Content percentages from the decomposed Co 2p spectra of CoSA/CoNP-NSDNC and Co-NSC

| Sample | Co^0^ | Co^2+^ | Co-N_x_ | Co^3+^ |
| --- | --- | --- | --- | --- |
| Co-NSC | 10.11 | 42.00 | 32.93 | 14.97 |
| CoSA/CoNP-NSDNC | 16.58 | 22.72 | 30.54 | 30.16 |

Table S7 Two-electron ORR performance of the various electrocatalysts, where the *E_0_* is the onset potential

| Catalyst | | Electrolytes | *E_0_* (V vs. RHE) | Selectivity （%） | Refs. |
| --- | --- | --- | --- | --- | --- |
| Non-noble-metal-based catalysts | CoSA/CoNP-NSDNC | 0.1 M HClO_4_ | 0.72 | 90 | This work |
|  | CoNOC | 0.1 M HClO_4_ | 0.57 | 95 | [S5] |
|  | Co_1_–NG(O) | 0.1 M HClO_4_ | 0.65 | 50 | [S6] |
|  | Co-N-C | 0.5 M H_2_SO_4_ | 0.78 | 80 | [S7] |
|  | Co-NC1 | 0.1 M HClO_4_ | 0.60 | 90 | [S8] |
| Noble-metal-based catalysts | PtHg_4_ | 0.1 M HClO_4_ | 0.58 | 96 | [S9] |
|  | Au/TiC | 0.1 M HClO_4_ | 0.35 | 87 | [S10] |
|  | Pd*_x_*-NC | 0.1 M HClO_4_ | 0.50 | 87 | [S11] |
|  | Pt/HSC | 0.1 M HClO_4_ | 0.71 | 95 | [S12] |
| Metal-free-based catalysts | g-N-CNHs | 0.1 M H_2_SO_4_ | 0.40 | 80 | [S13] |
|  | NCMK3IL50_800T | 0.5 M H_2_SO_4_ | 0.40 | 95 | [S14] |
|  | MCHS | 0.5 M H_2_SO_4_ | 0.40 | 70 | [S15] |

**Table S8** Raman characteristic properties of CoSA/CoNP-NSDNC samples prepared in different temperatures

| Catalyst | Intensity ratio of D and G | Area percentage of the D1 band | Area percentage of the D2 band | Area percentage of the D3 band | Area percentage of the D4 band | Area percentage of the G band | Area ratio of D3 and G |
| --- | --- | --- | --- | --- | --- | --- | --- |
| CoSA/CoNP-NSDNC-800 | 1.04 | 36.22 | 12.22 | 20.99 | 14.65 | 15.93 | 1.32 |
| CoSA/CoNP-NSDNC-900 | 1.02 | 36.52 | 14.16 | 18.25 | 14.96 | 16.11 | 1.13 |
| CoSA/CoNP-NSDNC-1000 | 1.05 | 38.29 | 13.66 | 15.85 | 17.92 | 14.28 | 1.11 |
| CoSA/CoNP-NSDNC-1100 | 0.98 | 42.75 | 15.99 | 11.52 | 16.2 | 13.55 | 0.85 |

Table S9 Content percentages from the decomposed N 1s spectra of CoSA/CoNP-NSDNC samples prepared in different temperatures

| Sample | Pyridinic-N | Co-N_x_ | Pyrrolic-N | Graphitic-N | Oxide-N |
| --- | --- | --- | --- | --- | --- |
| CoSA/CoNP-NSDNC-800 | 34.35 | 7.95 | 27.74 | 12.54 | 17.41 |
| CoSA/CoNP-NSDNC-900 | 30.18 | 14.15 | 15.11 | 21.78 | 18.79 |
| CoSA/CoNP-NSDNC-1000 | 8.94 | 8.16 | 39.59 | 29.1 | 14.21 |
| CoSA/CoNP-NSDNC-1100 | 12.42 | 3.61 | 22.98 | 31.92 | 29.06 |

Table S10 Comparing the property of CoSA/CoNP-NSDNC with recently reported materials as ORR catalysts

| Catalyst | Electrolyte | H_2_O_2_ production rates (mol g^-1^ h^-1^) | Faraday efficiency (%) | Refs. |
| --- | --- | --- | --- | --- |
| CoSA/CoNP-NSDNC | 0.1 M HClO_4_ | 4.2 | 90 | This work |
| CMK-3 | 0.1 M KOH | 0.561 | 70 | [S14] |
| PEI50CMK3_800T | 0.1 M K_2_SO_4_ | 0.57 | 65 | [S16] |
| N-C800 | 0.5 M NaCl | 0.63 | 79.8 | [S17] |
| NADE | 0.05M Na_2_SO_4_ | 0.22 | 66.8 | [S18] |
| CMK3-20s | 0.1 M K_2_SO_4_ | 0.83 | 78 | [S19] |
| ZPO/PC3 | 0.5 M NaCl | 3.52 | 95.9 | [S20] |
| FPC | 0.05M H_2_SO_4_ | 0.714 | 82.1 | [S21] |
| N-GA | 0.1 M H2SO4 | 0.107 | 70 | [S22] |

# Supplementary References

[S1] J. Park, Y. Nabae, T. Hayakawa et al., Highly selective two-electron oxygen reduction catalyzed by mesoporous nitrogen-doped carbon. ACS Catal. **4**, 3749-3754 (2014). <https://doi.org/10.1021/cs5008206>

[S2] G.-F. Han, F. Li, W. Zou et al., Building and identifying highly active oxygenated groups in carbon materials for oxygen reduction to H_2_O_2_. Nat. Commun. **11**, 2209 (2020). <https://doi.org/10.1038/s41467-020-15782-z>

[S3] C. Zhang, W. Shen, K. Guo et al., A Pentagonal Defect-Rich Metal-Free Carbon Electrocatalyst for Boosting Acidic O_2_ Reduction to H_2_O_2_ Production. J. Am. Chem. Soc. **145**, 11589-11598 (2023). <https://doi.org/10.1021/jacs.3c00689>

[S4] C. Zhang, J. Zhang, J. Zhang et al., Tuning coal into graphene-like nanocarbon for electrochemical H_2_O_2_ production with nearly 100% faraday efficiency. ACS Sustainable Chem. Eng. **9**, 9369-9375 (2021). <https://doi.org/10.1021/acssuschemeng.1c02357>

[S5] C. Tang, L. Chen, H. Li et al., Tailoring acidic oxygen reduction selectivity on single-atom catalysts via modification of first and second coordination spheres. J. Am. Chem. Soc. **143**, 7819-7827 (2021). <https://doi.org/10.1021/jacs.1c03135>

[S6] E. Jung, H. Shin, B.-H. Lee et al., Atomic-level tuning of Co-N-C catalyst for high-performance electrochemical H_2_O_2_ production. Nat. Mater. **19**, 436-442 (2020). <https://doi.org/10.1038/s41563-019-0571-5>

[S7] Y. Sun, L. Silvioli, N.R. Sahraie et al., Activity-selectivity trends in the electrochemical production of hydrogen peroxide over single-site metal-nitrogen-carbon catalysts. J. Am. Chem. Soc. **141**, 12372-12381(2019). <https://doi.org/10.1021/jacs.9b05576>

[S8] J. Gao, H.b. Yang, X. Huang et al., Enabling direct H_2_O_2_ production in acidic media through rational design of transition metal single atom catalyst. Chem **6**, 658-674 (2020). <https://doi.org/10.1016/j.chempr.2019.12.008>

[S9] S. Siahrostami, A. Verdaguer-Casadevall, M. Karamad et al., Enabling direct H_2_O_2_ production through rational electrocatalyst design. Nat. Mater. **12**, 1137-1143 (2013). <https://doi.org/10.1038/nmat3795>

[S10] S.K. Sahoo, Y. Ye, S. Lee et al., Rational design of TiC-supported single-atom electrocatalysts for hydrogen evolution and selective oxygen reduction reactions. ACS Energy Lett. **4**, 126-132 (2018). <https://doi.org/10.1021/acsenergylett.8b01942>

[S11] N. Wang, X. Zhao, R. Zhang et al., Highly selective oxygen reduction to hydrogen peroxide on a carbon-supported single-atom pd electrocatalyst. ACS Catal. **12**, 4156-4164 (2022). <https://doi.org/10.1021/acscatal.1c05633>

[S12] C.H. Choi, M. Kim, H.C. Kwon et al., Tuning selectivity of electrochemical reactions by atomically dispersed platinum catalyst. Nat. Commun. **7**, 10922 (2016). <https://doi.org/10.1038/ncomms10922>

[S13] X. Sheng, N. Daems, B. Geboes et al., N-doped ordered mesoporous carbons prepared by a two-step nanocasting strategy as highly active and selective electrocatalysts for the reduction of O_2_ to H_2_O_2_. Appl. Catal. B **176-177**, 212-224 (2015). <https://doi.org/10.1016/j.apcatb.2015.03.049>

[S14] Y. Sun, I. Sinev, W. Ju et al., Efficient electrochemical hydrogen peroxide production from molecular oxygen on nitrogen-doped mesoporous carbon catalysts. ACS Catal. **8**, 2844-2856 (2018). <https://doi.org/10.1021/acscatal.7b03464>

[S15] Y. Pang, K. Wang, H. Xie et al., Mesoporous carbon hollow spheres as efficient electrocatalysts for oxygen reduction to hydrogen peroxide in neutral electrolytes. ACS Catal. **10**, 7434-7442 (2020). <https://doi.org/10.1021/acscatal.0c00584>

[S16] Y. Sun, S. Li, Z.P. Jovanov et al., Structure, activity, and faradaic efficiency of nitrogen‐doped porous carbon catalysts for direct electrochemical hydrogen peroxide production. ChemSusChem **11**, 3388-3395 (2018). <https://doi.org/10.1002/cssc.201801583>

[S17] N. Wang, S. Ma, R. Zhang et al., Regulating N species in N‐doped carbon electro‐catalysts for high‐efficiency synthesis of hydrogen peroxide in simulated seawater. Adv. Sci. **10**, 2302446 (2023). <https://doi.org/10.1002/advs.202302446>

[S18] Q. Zhang, M. Zhou, G. Ren et al., Highly efficient electrosynthesis of hydrogen peroxide on a superhydrophobic three-phase interface by natural air diffusion. Nat. Commun. **11**, 1731 (2020). <https://doi.org/10.1038/s41467-020-15597-y>

[S19] Y.-L. Wang, S.-S. Li, X.-H. Yang et al., One minute from pristine carbon to an electrocatalyst for hydrogen peroxide production. J. Mater. Chem. A **7**, 21329-21337 (2019). <https://doi.org/10.1039/C9TA04788C>

[S20] Q. He, S. Zhan, J. Li et al., ZrP_2_O_7_/P-doped carbon: A pathway to enhanced 2e^−^ ORR activity in seawater environments. Appl. Surf. Sci. **655**, 159536 (2024). <https://doi.org/10.1016/j.apsusc.2024.159536>

[S21] K. Zhao, Y. Su, X. Quan et al., Enhanced H_2_O_2_ production by selective electrochemical reduction of O_2_ on fluorine-doped hierarchically porous carbon. J. Catal. **357**, 118-126 (2018). <https://doi.org/10.1016/j.jcat.2017.11.008>

[S22] Y. Zhang, M. Melchionna, M. Medved et al., Enhanced on-site hydrogen peroxide electrosynthesis by a selectively carboxylated N‐doped graphene catalyst. ChemCatChem **13**, 4372-4383 (2021). <https://doi.org/10.1002/cctc.202100805>
